# PepTCR-Net: prediction of multi-class antigen peptides by T-cell receptor sequences with deep learning

**DOI:** 10.1093/bib/bbaf351

**Published:** 2025-07-24

**Authors:** Phi Le, Leah Ung, Hai Yang, Anwen Huang, Tao He, Peter Bruno, David Y Oh, Bridget P Keenan, Li Zhang

**Affiliations:** Department of Medicine, University of California San Francisco, 550 16th Street, San Francisco, CA 94158, United States; Department of Medicine, University of California San Francisco, 550 16th Street, San Francisco, CA 94158, United States; Helen Diller Family Comprehensive Cancer Center, University of California San Francisco, 1450 3rd St. San Francisco, CA 94158, United States; Department of Medicine, University of California San Francisco, 550 16th Street, San Francisco, CA 94158, United States; University of California Berkeley, 110 Sproul Hall, Berkeley, CA 94720, United States; Department of Mathematics, San Francisco State University, 1600 Holloway AvenueSan Francisco, CA 94132, United States; Helen Diller Family Comprehensive Cancer Center, University of California San Francisco, 1450 3rd St. San Francisco, CA 94158, United States; Department of Urology, University of California San Francisco, 1450 3rd StreetSan Francisco, CA 94158, United States; Department of Medicine, University of California San Francisco, 550 16th Street, San Francisco, CA 94158, United States; Helen Diller Family Comprehensive Cancer Center, University of California San Francisco, 1450 3rd St. San Francisco, CA 94158, United States; Department of Medicine, University of California San Francisco, 550 16th Street, San Francisco, CA 94158, United States; Helen Diller Family Comprehensive Cancer Center, University of California San Francisco, 1450 3rd St. San Francisco, CA 94158, United States; Department of Medicine, University of California San Francisco, 550 16th Street, San Francisco, CA 94158, United States; Helen Diller Family Comprehensive Cancer Center, University of California San Francisco, 1450 3rd St. San Francisco, CA 94158, United States; Department of Epidemiology & Biostatistics, University of California San Francisco, 550 16th Street, San Francisco, CA 94158, United States

**Keywords:** antigen peptides, Atchley factor, encoder–decoder, graph embedding, neural networks, T-cell receptor network

## Abstract

Predicting T-cell receptor (TCR) recognizing antigen peptides is crucial for understanding the immune system and developing new treatments for cancer, infectious and autoimmune diseases. As experimental methods for identifying TCR–antigen recognition are expensive and time-consuming, machine-learning approaches are increasingly used. However, existing computational tools often struggle with generalization due to limited data and challenges in acquiring true non-recognition pairs and rarely integrate multiple biological features into unified frameworks. To address these challenges, we propose a two-step framework for predicting TCR–antigen recognition. The first step focuses on feature engineering: neural network-based embeddings of letter-based TCR and peptide sequences inspired by language models, and categorical encoding of Human Leukocyte Antigen types and Variable/Joining genes. In the second step, we built a prediction model to assess the likelihood of TRC–antigen recognition by a Bayesian Feedforward Neural Network. We trained and validated the framework using large public databases. Our results demonstrate that our advanced feature engineering delivers strong predictive performance both internally and externally. We applied the framework to a real-world case for predicting whether specific TCRs can recognize SARS-CoV-2 epitope peptides, demonstrating that our framework can function as a *de novo* TCR–antigen prediction tool applicable to infectious diseases.

## Introduction

T cells are specialized immune cells that play a crucial role in killing pathogens, such as virus-infected cells, certain bacteria, and cancer cells, through cell-mediated immunity. T cells identify infected or abnormal host cells via the T-cell receptor (TCR), a surface protein that recognizes antigenic peptides. Typically, antigens are foreign substances that the immune system recognizes as non-self and potentially harmful but can also be self-antigens in the context of autoimmunity and malignancy. The primary function of the immune system is to detect and respond to antigens to protect the body from infections and other threats by recognizing pathogen-derived peptides presented in the context of Major Histocompatibility Complex (MHC) molecules on the infected cell surface. Understanding the recognitions between TCRs and antigen peptides helps us grasp the dynamics of immune responses and how the immune system identifies and targets threats. The diversity of TCRs generated through a highly specialized process in the immune system called V(D)J recombination enables our immune system to identify an almost unlimited number of antigen peptides. However, this diversity poses a major challenge in predicting whether a given TCR can recognize a specific antigen peptide. Additionally, there is known degeneracy of TCR–peptide–MHC interactions such that a given TCR can recognize multiple structurally unrelated peptides [1]. Accurate prediction of T-cell activation based on antigen peptide and TCR sequences would significantly impact research fields across infectious diseases, autoimmunity, vaccine development, and cancer immunology.

To study the architecture of the TCR repertoire, researchers have analyzed the similarity across TCR sequences by constructing a TCR network based on distance matrices [2]. Several approaches have focused on transforming letter-based TCR amino acid sequences into numerical vectors. For example, Net-TCR-2.0 [3] used the BLOSUM50 [4] score matrix which quantifies the probability of the biological meaning of amino acid in an alignment. ImRex [5] used physicochemical properties (mass, hydrophobicity, hydrophilicity, and isoelectric point). Tessa [6] developed a deep learning autoencoder model to decode TCR sequences using Atchley factors [7], while ATM-TCR [8] used a one-hot encoding approach [9]. Most of those methods, except Tessa, were tightly coupled with their prediction models and did not consider the positions of the amino acids in the sequences, which is the key factor in determining protein structure and its folding status. We propose a new method based on the ideas from Natural Language Processing [10] to find representations of letter-based amino acid sequences using Atchley factors together with Positional Encoding (PE). Positional encoding can differentiate the same amino acid at different positions within a sequence while keeping the correlation of the same amino acid.

Furthermore, when the TCR recognizes the peptide, structural interactions occur between a TCR and an antigen peptide [11]. To capture meaningful representations of TCRs based on this recognition information, we propose using the Encoder–Decoder (ED) technique that learns a shared latent space capable of reconstructing both TCR and peptide sequences when they form a functional pair. The ED model [12, 13] is a method that converts data from one domain to another through this latent representation, commonly used for language translation. In our approach, the ED model maps both TCR and peptide sequences into a shared latent space from which both can be reconstructed. Researchers have used unsupervised approaches such as similarity clustering (e.g. NAIR [2]) to study TCR-antigen peptide recognition since TCRs with highly similar sequences often recognize the same antigen peptide. Therefore, we further built a new model, Node Embedding (NE), which integrates the TCR sequence similarity with our ED-derived latent representation by using a Graph Neural Network [14] to obtain another representation of TCR.

Various studies [8, 15, 16] have been conducted to predict TCR–antigen peptide recognition. These TCR–Peptide pair methods [8, 15, 16] often rely on algorithmically generated negative labels rather than experimentally validated non-recognition data, which can introduce significant bias and lead to mislabeled examples. To overcome these issues, we propose a new approach for identifying TCRs that can recognize specific peptides of interest. Given that a TCR may recognize multiple interested peptides or none at all, we apply an uncertainty-sensitive model, the Bayesian Feedforward Neural Network (BFNN) [17–19], which can provide probabilistic insights into TCR–peptide recognition. Our model is trained and tested on the three largest publicly available, wet lab-verified databases (i.e. IEDB [20], VDJdb [21], McPAS-TCR [22]), therefore, avoiding using synthesized negative labels. We demonstrate the practical utility of the framework by applying it to a COVID dataset [23] to predict if specific TCRs can recognize SARS-CoV-2 epitope peptides. This field has demonstrated practical impact in various real-world immunological applications, including measuring clonal expansion of T cells, identifying immune-responsive TCRs in emerging diseases, and sorting T cells specific to neoantigens [24, 25], a key step in adoptive cell transfer-based immunotherapy [26], identifying the immune-responsive TCRs of new diseases. For example, researchers [27] have utilized TCR–peptide prediction models, such as NetMHCpan [28], to verify MOC1-specific neoantigens.

## Methods

**Figure 1:**
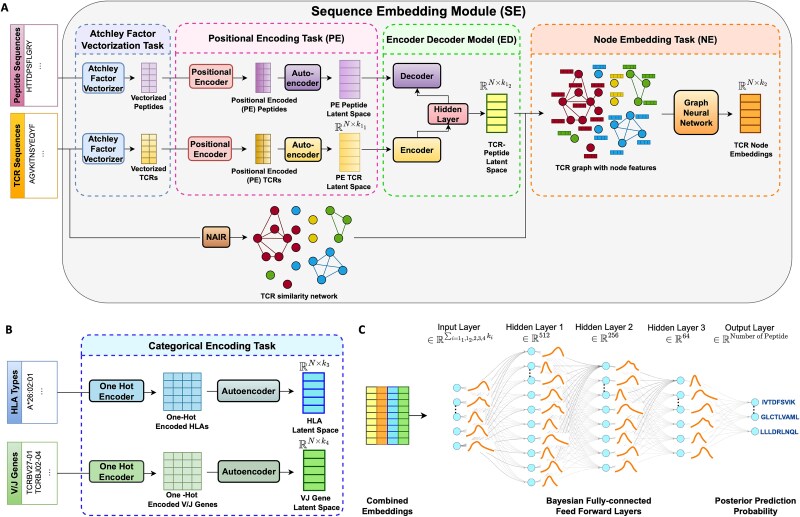
The workflow of pep-TCR net (A) architecture of the TCR and peptide amino acid SE pipeline. The pipeline introduces three different embedding approaches: PE task, ED module, and NE module. We first use an Atchley factor vectorizer to generate numerical vectors of TCR sequences and peptide amino sequences. Next, in the PE task, we apply an autoencoder to normalize the positionally encoded sequences and filter out potential sequencing noise and extract the final layer of the encoder block (i.e. TCR latent space and peptide latent space). In ED module, we input the TCR latent space into the encoder block, then use the peptide latent space as the output of the decoder block of the ED model and extract the final layer of the encoder block to create the TCR–peptide latent space. For NE module, we first generate an undirected graph of the TCR similarity network by NAIR R package and use the TCR–peptide latent space as node features. We then use a semi-supervised graph neural network to obtain TCR NEs. (B) Categorical encoding module for HLA types and VJ genes. This module accepts HLA types or VJ genes as input, then employs a one hot encoder to generate one-hot encoded features. An autoencoder is then used to minimize noise and reduce dimensionality. The final layer of the encoder block in the model is extracted to generate the HLA and VJ gene latent spaces. (C) Bayesian feed forward prediction. The inputs for the model are TCR sequences which are the output of either PE, or ED or PE + NE or ED + NE, with the flexibility to include HLA type and VJ genes which are the outputs of categorical encoding module. The Bayesian feedforward neural network (BFNN) model is designed with three fully connected hidden layers, each having a probability distribution assigned to its weights. To mitigate overfitting, normalization and dropout layers are incorporated after each fully connected layer. The model’s output is a marginal predictive distribution, which is a multivariate posterior distribution based on the predictor input and the trained weights.

Our pipeline PepTCR-Net consists of two steps: feature engineering, which includes two modules (Fig. 1A and B), and prediction, which focuses on building a prediction model for TCR–peptide recognition (Fig. 1C).

### Part I feature engineering

#### Atchley factor vectorization task

Similar to Tessa [6], we use Atchley Factor Vectorization to transform an amino acid sequence into a numerical vector using Atchley factors [7], which represent ~500 different amino acid properties into five main factors (i.e. polarity, secondary structure, molecular volume, codon diversity, and electrostatic charge). In addition to these five, we include the average of the five factors as the 6th factor. This transforming process includes several steps, with the details in the Supplementary Document and Supplementary Fig. 1.

#### SE module

In the following subsections, we will introduce three advanced Deep Learning models (Fig. 1A) designed to generate TCR representations based on the Atchley factor encoding of amino acid sequences: (i) PE: to incorporate the position of individual amino acids in sequences (Supplementary Fig. 2A), (ii) ED: to captures feature-rich information of TCR-antigen recognition (Supplementary Fig. 2B), and (iii) NE of TCR Sequences: to use the similarity of letter-based TCR sequences (Supplementary Fig. 2C).

#### Positional encoding

The same amino acid in different sequence positions can play different roles in the protein structure. For example, the *A* at position 2 in the sequence C*A*SSQGL*A*YEQFF plays a different role than the *A* at position 8. To address this, we introduce a PE [10] method (Supplementary Fig. 2A) to enhance the TCR sequence embeddings (SEs) by accounting for the biological importance of amino acid positions. To factor in both the position of each amino acid and the positional correlations of the identical amino acid at different positions, we add small values of $sine\left(\frac{i}{1000}\right)$ for the odd position $i$ and $1-\cos \left(\frac{j}{1000}\right)$ for the even position $j$. The output of positional embedding can be used directly as input for the prediction model or passed to the ED model described below.

Since the maximum lengths of TCR and peptide sequences differ, sequencing techniques and the zero-padding used in Atchley factor vectorization may produce uncontrolled noise. To mitigate this, we build autoencoders for TCRs and peptides to normalize the numerical vectors produced by PE to reduce dimensionality and remove potential sequencing artifacts. The output from the final layer of the encoder is the latent space and is considered as a TCR representation, which retains the critical information and inherent structural features of the original input. To make sure the sequence lengths are consistent across the data, we empirically determined that a latent space with dimension 121 offers a suitable balance between resolution and generalizability.

#### ED model

We propose an ED model to better decode TCR and peptide sequences by using the known relationships between TCR and antigens from established databases. An ED model [12, 13] is commonly used to learn transformation between domains and typically consists of two main components: an encoder, which maps input data to a latent space, and a decoder, which reconstructs output data from this latent representation. In our framework, the encoder source is TCR sequences, and the decoder aims to reconstruct the corresponding antigen peptide recognized by the TCR (Fig. 1A). Both TCR and peptide representation are initially derived from the PE autoencoder model. We then extract the latent space, the output of the Encoder layer from the ED model (Supplementary Fig. 2B) as the representation of the TCR sequence. Our ED model is trained on the three largest curated datasets IEDB [20], VDJdb [21] and McPAS-TCR [22] that contain experimentally validated TCR–peptide recognitions.

#### NE of TCR sequences

TCRs with high similarity are more likely to recognize the same antigen peptides. Therefore, to leverage this relationship, we integrate the TCR sequence similarity structure and the ED representations using a method called NE. We first construct a similarity network of TCR sequences using the NAIR R package [2], where each node denotes a TCR sequence, and edges connect nodes with a Levenshtein distance of zero. To capture the topological structure of the network and the learned ED representations, we employ a semi-supervised approach to train the GraphSage model [29]. The TCR similarity network serves as the graph input, and the TCR–peptide latent space vectors are used as node features during training. Once trained, this NE model generates NEs that can be used as input for prediction tasks.

#### Categorical encoding

In addition to TCR sequences, we also include VJ gene information and Human Leukocyte Antigen (HLA) types in the prediction model. Since both are categorical variables, we consider one hot encoding [30], which converts categorical variables into indicator variables (Supplementary Fig. 3). Each category is represented by separate variable with values of 0 or 1, indicating its presence or absence, respectively. We further use an autoencoder to mitigate the high dimensionality and sparsity introduced by one-hot encoding.

### Part II prediction model by Bayesian feedforward neural network

Our prediction model aims to predict if TCRs can recognize known antigen peptides, acknowledging that some TCRs may recognize multiple peptides—or none. To incorporate the prediction uncertainty for TCR–peptide recognition, we apply the BFNN [17–19] (Fig. 1C). Unlike classical neural network, the Bayesian model infers learning parameter weights and biases based on the posterior distributions [18] rather than fixed weights. Let $y=f\left(x,\theta \right)$ be the predicted output (i.e. the peptide class) given the input x (e.g. TCR, VJ gene, HLA types) and the parameters $\theta$. The parameters $\theta$ are estimated by using the Bayesian posterior distribution


$$ p\left(\theta |\mathrm{D}\right)\sim p\left(D|\theta \right)p\left(\theta \right), $$

where $p\left(\theta \right)$ is a standard normal prior and $D=\left(X,Y\right)$is the training data. Given a new input x*, the predictive distribution of the output $y\ast$ is


$$ p\left(y\ast |x\ast, D\right)=\int p\left(y\ast |x\ast, \theta \right)p\left(\theta |D\right) d\theta $$

We extract 200 samples from the predictive distribution to estimate the probability of TCR–peptide recognition. The peptide with the highest median sampling probability is identified as the predicted peptide. The spread of probabilities across classes reflects prediction uncertainty. To mitigate the class imbalance, we employed the sample weight learning technique [31].

## Results

### Prediction performance on in-distribution data

**Figure 2:**
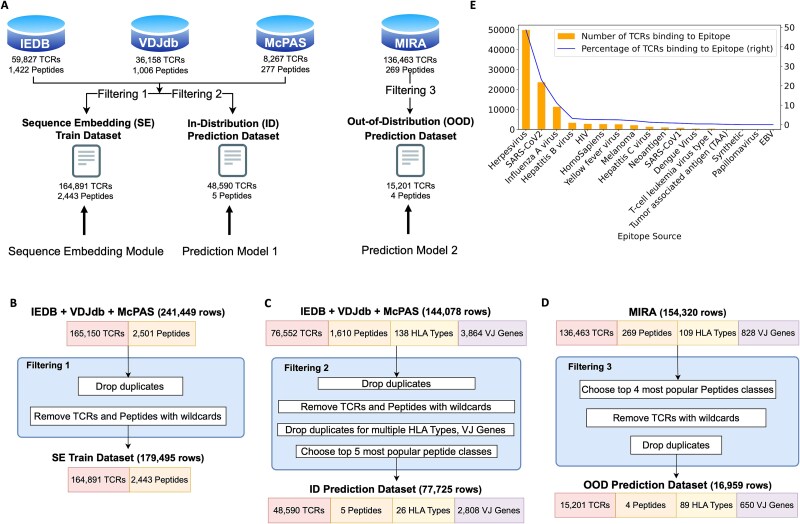
Overview of data curation (A) data curation workflow. Based on three public databases (IEDB, VDJdb and McPAS), we create the sequencing embedding (SE) train dataset (used to train SE module) and the ID prediction dataset (used to train prediction model 1) using filtering strategy 1 and 2, respectively. We also generate the out-of-distribution prediction dataset (used to train prediction model 2) from the MIRA public database. (B) SE train dataset. Starting with 165 150 TCRs and 2501 peptide sequences (with 241 449 TCR–peptide pairs) from IEDB, VDJdb, and McPAS, after removing TCR or peptide sequences that contain wildcard letters by filtering 1, we have 164 891 TCRs and 2443 peptides (with 179 495 TCR–peptide pairs). (C) ID prediction dataset. Starting with the entries with 76 552 TCRs, 1610 peptide sequences, 136 HLA types, and 3864 VJ genes (with 144078 rows) from the IEDB, VDJdb, and McPAS datasets, after dropping the duplicates and the TCRs binding to multiple antigen peptides, TCR and peptide sequences with wildcard characters as well as any HLA types and VJ genes that contain more than one categories by filtering 2, we have 76 445 TCRs, 1601 peptides, 130 HLA types, and 3860 VJ genes (with 127 635 rows). (D) Out-of-distribution prediction dataset. Starting with the entries with 136 463 TCRs, 259 peptide sequences, 109 HLA types, and 828 VJ genes (with 154 320 rows) from the MIRA dataset, filtering 3 selects the four most popular SARS-CoV-2 peptide classes, eliminating any TCR sequences with wildcards, and removing the duplicates from the dataset. There are 15 201 TCRs, 4 peptide classes, 89 HLA types, and 650 VJ genes (16 959 rows). (E) Distribution of peptide sources in the SE train dataset. The bar plot displays the number of unique TCRs binding to epitope (left y-axis) and the percentage of unique TCRs binding to epitope (the right y-axis) for different types of human viruses (x-axis), representing different peptide sources.

Our framework, PepTCR-Net, integrates feature engineering and prediction modeling. To ensure the robust performance of the framework, we trained and validated these components under three setups (Fig. 2A): SE training (Fig. 2B), In-Distribution (ID) prediction (Fig. 2C) and Out-of-Distribution (OOD) prediction (Fig. 2D). Supplementary Table 1 summarizes the three datasets.

For SE training, we obtained the curated peptide-TCR recognizing records from three largest publicly available databases: IEDB [20], VDJdb [21], and McPAS-TCR [22]. From these datasets, we retained entries for *Homo sapiens* and CDR3 beta chains. The SE training dataset contains 179 495 unique TCR–peptide pairs (164 891 unique TCRs and 2443 unique peptides). Figure 2E presents the distribution of peptide sources in this dataset. First, using the SE training data (Fig. 2B), we trained the ED model to obtain representations for TCR–peptide pairs and then used those representations to train the NE model to obtain TCR similarity-based network representations.

For ID prediction, we again used the same three databases but focused on the five most frequent peptides and also extracted HLA type and VJ gene information as additional predictive features. The ID dataset includes 77 725 entries with 48 590 unique TCRs (KLGGALQAK (25 683 entries and 14 515 TCRs), YVLDHLIVV (17 038 entries and 8410 TCRs), GLCTLVAML (12 824 entries and 7514 TCRs), GILGFVFTL (11 895 entries and 8682 TCRs), and NLVPMVATV (10 285 entries and 9469 TCRs)), 26 unique HLA types, and 2808 unique VJ genes. Supplementary Table 2 presents the HLA type distribution across these five peptides. Since 71% of TCR–peptide pairs were used in the SE training (Supplementary Table 1), this setup evaluates the model’s internal generalization. The features derived from the feature engineering modules (i.e. PE, ED, NE, HLA, and VJ) are the inputs. The ID data were randomly divided into training (72%), validation (8%), and testing (20%).

**Figure 3:**
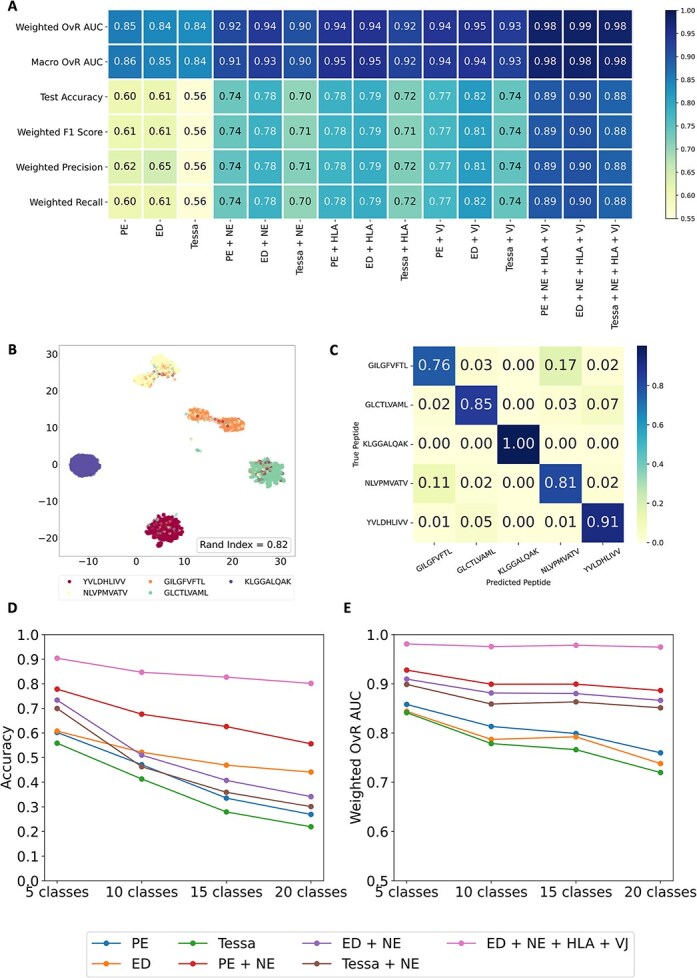
Performance evaluation of pep-TCR net on ID prediction dataset (A) heatmap depicting the prediction performance of the top five most frequent peptides based on the Bayesian feed forward (BFF) model with different prediction inputs. The prediction performance was evaluated based on six metrics (by row): Weighted one-vs-rest (OvR) AUC, macro OvR AUC, test accuracy, weighted F1 score, weighted precision, and weighted recall. this evaluation was carried out for fifteen variations of inputs for the prediction model (by column): Encoded TCR sequences alone (PE, ED, and Tessa); encoded TCR sequences combined with NEs, i.e. PE + NE, ED + NE and Tessa+NE; encoded TCR sequences combined with HLA types, i.e. PE + HLA, ED + HLA and Tessa+HLA; encoded TCR sequences combined with VJ genes, i.e. PE + VJ, ED+ VJ and Tessa+ VJ; encoded TCR sequences combined with NE, HLA types, and VJ genes simultaneously, i.e. PE + NE + HLA + VJ, ED + NE + HLA + VJ and Tessa+NE + HLA + VJ. (B) UMAP plot of the combined embeddings of ED + NE + HLA + VJ. We applied the supervised UMAP using the predicted label information on the combined ED + NE + HLA + VJ dataset for the top five most frequent peptides based on the BFF model. the UMAP was color-coded based on the five most frequent peptides. The Rand index (RI) was used to assess the consistency of clustering with the true peptide groups. (C) the multi-class confusion matrix of the top five most frequent peptides with the combined embeddings of ED + NE + HLA + VJ as the prediction input. Rows and columns represent the true and predicted peptides. The heat indicates the mean predicted score over the TCRs corresponding to the true and predicted peptides. (D) Line plot of the prediction accuracy to predict different number of classes based on the BFF model with different prediction inputs. Each line represents the prediction accuracy (y-axis) across different number of classes (x-axis): 5, 10, 15, and 20 for one embedding approach, including PE, ED, Tessa, PE + NE, ED + NE, Tessa+NE and ED + NE + HLA + VJ. (E) Line plots of the weighted AUC to predict different number of classes for various combinations of embeddings based on the BFF model with different prediction inputs. Each line represents the weighted AUC (y-axis) across different number of classes (x-axis): 5, 10, 15, and 20 for one embedding approach: PE, ED, Tessa, PE + NE, ED + NE, Tessa+NE and ED + NE + HLA + VJ.

We calculated the performance metrics based on 200 sampling from the posterior distribution, we repeated 30 times to obtain the mean and variance of each performance metric. Figure 3A summarizes the mean prediction performance based on different input combinations and Supplementary Table 3 summarizes the variance of prediction performance. Models using only sequence-based features (i.e. PE, ED, and Tessa) perform comparably. As expected, the full models, adding the HLA or VJ gene achieve the best performance with AUC near 1, and accuracy, F1 score, precision, and recall are all close to 0.9. Notably, incorporating NE significantly improves performance compared to using PE, ED, and Tessa alone, e.g. improving accuracy by at least 14% and AUC by at least 6%. These findings highlight the power of NE in integrating TCR sequence similarity with TCR embedding, therefore improving the prediction performance. NE also provides a robust alternative when the HLA or VJ gene is incomplete or missing.

To visualize the model's performance, we applied the supervised UMAP [32] to the combined embeddings (ED + NE + HLA + VJ), color-coded by the true label based on the top five most frequent peptides (Fig. 3B). The resulting clustering had a rand index [33] of 0.82, indicating the model's excellent performance. The heatmap (Fig. 3C) shows the average prediction score for each true-predicted peptide pair. The average prediction score was taken over all TCRs within each true-predicted peptide pair. As expected, the diagonal values of the matrix are very close to 1, indicating high confidence and accuracy in predictions. In contrast, off-diagonal values capture uncertainty and occasional misclassifications.

We also used the violin plots to visualize the posterior prediction probability distribution of the top five most frequent peptides based on the BFNN [18] with ED + NE + HLA + VJ as the inputs (Supplementary Fig. 4). For each true peptide, five TCRs were randomly selected to illustrate the posterior prediction distribution (based on 200 samplings) across predicted peptides. The predicted label was determined based on the highest prediction probability within each panel. For example, in the first panel of the first row, TCR CASCSLTGSGETLYF is correctly predicted as recognizing its true peptide, YVLDHLIVV, with a median posterior prediction probability of 1, while the other peptides show a median posterior prediction probability of 0. In contrast, in the third panel of the third row, the model incorrectly predicts NLVPMVATV as the most likely peptide because its posterior probability is much higher than the true peptide GILGFVFTL. Although overall mispredicted rates are low, some correct predictions (e.g. panels 1 and 4 in the third row) showed moderate confidence, with the median posterior probabilities of 0.67 and 0.69, respectively. These results highlight the value of Bayesian modeling, offering not only accurate predictions but also valuable insight into prediction confidence and uncertainty.

### Consistent performance across different numbers of classes

We evaluated model performance in multi-class classification settings with 5 to 20 classes and found that, as expected, accuracy and AUC declined with more classes. Overall, our models, especially those including NE, show robustness even as the number of classes increases (Fig. 3D-E, Supplementary Figs. 5). The supplementary document provides a more detailed description of the results.

### Prediction performance on out-of-distribution data

Next, we evaluate the robustness of SE and prediction models using external data, the MIRA dataset [23], a curated SARS-CoV-2 dataset with both TCRs and verified antigen peptides (Fig. 2D). We focused on four peptide classes associated with SARS-CoV-2 epitopes, verified through wet lab experiments [34–36] (Fig. 2D). LSPRWYFYY and SPRWYFYYL were treated as one class due to their shared TCR recognition, as were AYKTFPPTEPK and KTFPPTEPK. Supplementary Table 4 presents the HLA type distribution across these four peptides. Notably, only ~4% of TCR–peptide pairs in the MIRA dataset were seen during SE training (Supplementary Table 1), making this an OOD test. We randomly split the data into training (72%), validation (8%), and test (20%) sets. We trained the model on the training set, tuned it using the validation set to prevent overfitting, and evaluated it in the test set for generalization performance.

**Figure 4:**
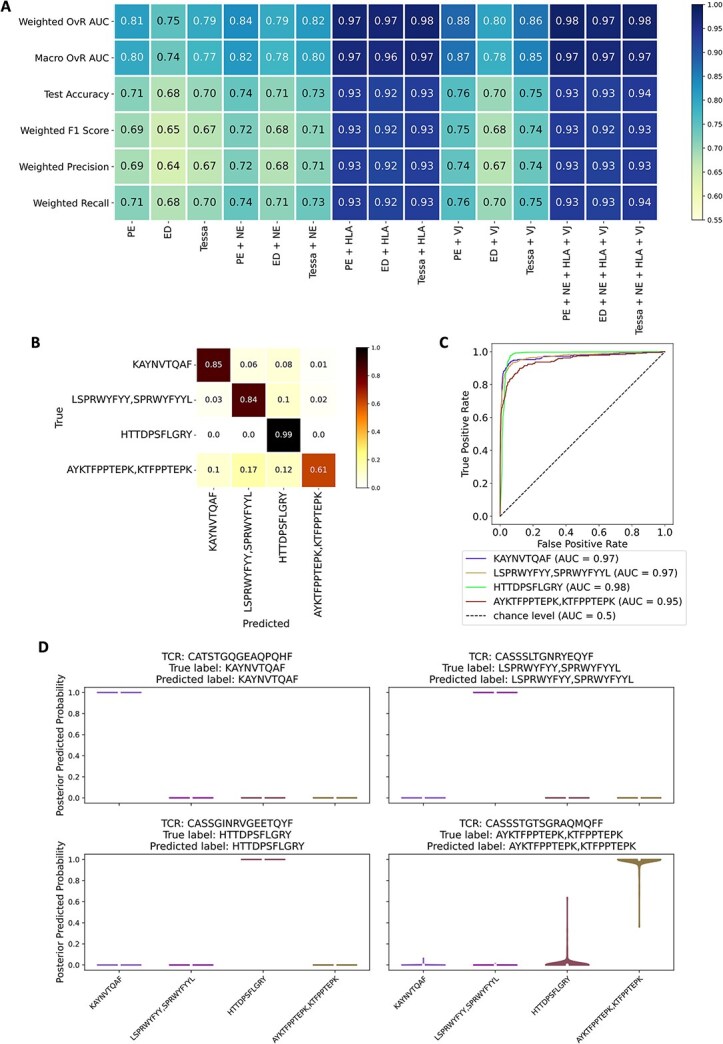
Performance evaluation of pep-TCR net on OOD prediction dataset. (A) Heatmap depicting the prediction performance of the Bayesian feed forward (BFF) model with different prediction inputs. The Bayesian model was evaluated based on six metrics (by row): Weighted one-vs-rest (OvR) AUC, macro OvR AUC, test accuracy, weighted F1 score, weighted precision, and weighted recall. this evaluation was carried out for fifteen variations of inputs for the prediction model (by column): Encoded TCR sequences alone (PE, ED, and Tessa); encoded TCR sequences combined with NEs, i.e. PE + NE, ED + NE and Tessa+NE; encoded TCR sequences combined with HLA types, i.e. PE + HLA, ED + HLA and Tessa+HLA; encoded TCR sequences combined with VJ genes, i.e. PE + VJ, ED+ VJ and Tessa+ VJ; encoded TCR sequences combined with NE, HLA types, and VJ genes simultaneously, i.e. PE + NE + HLA + VJ, ED + NE + HLA + VJ and Tessa+NE + HLA + VJ. (B) the multi-class confusion matrix of the prediction accuracy for the four peptide classes with ED + NE + HLA + VJ as the prediction input. Rows and columns represent the true and predicted peptides. The heat indicates the accuracy corresponding to the true and predicted peptides. (C) OvR ROC curves for the four peptide classes with the corresponding AUC scores. Each curve represents one of the peptide classes. (D) Violin plots of the posterior prediction probability. For each true peptide (each panel), one TCR was randomly selected, where x-axis represents four predicted peptides, the y-axis plots the distribution of the posterior prediction probability (based on 200 sampling) of the TCR for each predicted peptide. The predicted label is based on the highest prediction probability. For example, for top left panel, TCR CASSLFVGSGGVREKLFF is predicted to bind to peptide KAYNVTQAF which is the true binding peptide.

As observed in the ID dataset results, adding NE achieves better performance than using PE, ED, and Tessa alone, and the full model (TCR+ HLA+ VJ) performs the best (Fig. 4A). Supplementary Table 5 summarizes the variance of prediction performance. We further evaluated multi-class classification performance on this OOD dataset using the ED + NE + HLA + VJ feature combination by a confusion matrix. Figure 4B shows that the model accurately classified TCRs recognizing the correct peptides, achieving 99% accuracy for peptide HTTDPSFLGRY. The model also can handle the minor class KAYNVTQAF (7.5% of the data) well, with 85% recall. For the smallest class (7% of data), we obtained a recall of 61% with misclassifications spread relatively evenly across the other classes. This might be explained by the assumption that peptides AYKTFPPTEPK and KTFPPTEPK may share structural features that make them more easily recognized by TCRs, potentially contributing to higher misclassification rates. In addition, the OvR ROC (37)curves demonstrate strong class separation, with AUCs greater than 0.95 (Fig. 4C).

To assess model uncertainty, we randomly selected one TCR for each true peptide (each panel in Fig. 4D) and plotted the posterior prediction probability distribution (based on 200 samplings) across all four peptide classes. The peptide class with the highest median posterior prediction probability across 200 samples was considered as the predicted label. For example, in the top left panel, since the median posterior prediction probability of peptide KAYNVTQAF is 1 while the median probabilities for the rest of the three peptide classes are all 0, TCR CASSLFVGSGGVREKLFF is predicted to recognize KAYNVTQAF which is also the true peptide. While the Bayesian prediction model introduces some prediction uncertainty (as shown in the bottom right panel in Fig. 4D), the overall prediction performance in the external data is still stable and reliable.

### Application to real-world data

**Figure 5:**
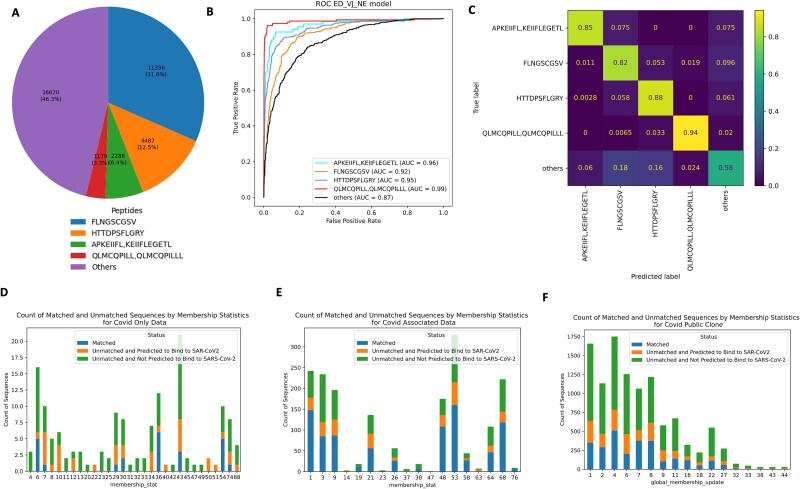
Application to real-world data (A) pie chart of distribution of the four SARS-CoV-2 epitope peptide classes in MIRA data. (B) OvR ROC curves for the four peptide classes and ‘other’ class with the corresponding AUC scores in MIRA data with ED + NE+ VJ as the prediction input. Each curve represents one of a class. (C) the multi-class confusion matrix of the prediction accuracy for the four peptide classes and ‘other’ class with ED + NE+ VJ as the prediction input in MIRA data. Rows and columns represent the true and predicted peptides. The heat indicates the accuracy corresponding to the true and predicted peptides. (D, E, F) the bar charts of the number of TCRs for COVID-associated clusters, COVID-only clusters, and public clusters, respectively. Each bar stands for a single TCR cluster. The bar heights represent the number of the different categories of TCRs, i.e. the TCRs which exactly matched with MIRA data (blue), the TCRs which are unmatched with MIRA data but predicted to recognize one of the four peptide classes (orange) and the TCRs which are unmatched with MIRA data and not predicted to recognize any of the four peptide classes (green).

In this section, we demonstrate how to apply the proposed framework to real-world data. In our recent study [2], we identified three types of TCR clusters: COVID-only clusters, COVID-associated clusters and public clusters with a total of 19 731 TCRs (listed in Supplementary Tables 3, 4, and 5 in [2]) from a study of European subjects [37]. The dataset included three groups collected during the early stage of the pandemic in Europe: individuals who recovered from COVID-19, those with active infections, and age-matched healthy donors. We applied PepTCR-Net to predict whether any of these TCRs can recognize four confirmed SARS-CoV-2 epitope peptide classes, i.e. 'FLNGSCGSV', 'HTTDPSFLGRY', 'APKEIIFL, KEIIFLEGETL', 'QLMCQPILL, QLMCQPILLL’. We trained the models on the curated SARS-CoV-2 MIRA dataset [23]. Since it could happen that a given TCR may not recognize any of the peptides listed above, we grouped all other peptides from the MIRA dataset that are not the four peptide classes under an "others" class. However, due to the large amount of TCRs in the “others” class, we only kept up to 100 TCRs per peptide within the “others” class. Figure 5A presents the distribution of the five classes.

We then applied the feature engineering steps to extract PE, ED, and NE representations of TCR sequences and VJ genes, as shown in Figs. 1A and 1B. Since we do not have valid HLA information for the European cohort, we trained the prediction model using ED + NE + VJ. We obtained the OvR weighted AUC [38] score of 0.91, macro-AUC [38] score of 0.93, the accuracy of the TCR exact match of 0.74, weighted F1 score of 0.74, weighted Precision of 0.77, and weighted Recall of 0.74. Figs. 5B and 5C also present the ROC curves for each peptide class and the accuracy confusion matrix of the training data. The model performance is similar to the results observed in ID and OOD settings.

Next, we applied the trained model to predict these 19 731 TCRs from the European cohort. To ensure prediction confidence, we only assigned the TCRs with prediction scores greater than 0.5 to the corresponding peptide classes and labeled those with scores less than 0.5 as the ‘others’ class. Among the 148 TCRs in the identified COVID-only TCR clusters, 24 TCRs exactly matched with the MIRA dataset (the blue bars in Fig. 5D), PepTCR-Net predicted 36 TCRs (out of the remaining 124 unmatched TCRs) likely recognize one of the four classes (orange bars in Fig. 5D). In the COVID-associated TCR clusters (Fig. 5E), 281 (out of total 921 unmatched) TCRs, and in public TCR clusters (Fig. 5F), 1861 (out of total 8180 unmatched) TCRs were predicted to recognize one of the four classes.

### Comparison with existing methods

As noted above, NE offers a robust alternative to enhance the prediction performance when the HLA type information is unavailable, or the VJ gene annotations are incomplete or missing. We tested the five most common peptides from the ID dataset to evaluate how adding NE to TCR embeddings generated by PE, ED, and Tessa impacted clustering quality. The results (Supplementary Fig. 7 and Supplementary Document) show that incorporating NE improves the clustering structure. We also compared PepTCR-Net with existing models ERGO-II [15], ATM-TCR [8], and Panpep [16] and found that PepTCR-Net consistently outperforms them (Supplementary Fig. 7 and Supplementary Document). We further extended the comparisons to include traditional machine learning models (Random Forest, Support Vector Machines, and XGBoost) [39]. While our BFNN models achieved comparable performance across multiple evaluation metrics (Supplementary Fig. 8 and Supplementary Document), its probabilistic nature offers greater interpretability, allowing researchers to better understand uncertainty and model behavior, features not provided by standard classifiers.

## Conclusion and discussion

This study presents PepTCR-Net, a novel framework for predicting TCR–peptide recognition using biologically informed and modular feature engineering. Our method transforms letter-based TCR amino acid sequences into numerical vector representations (Fig. 1A), capturing molecular structure, physicochemical properties, electrostatic charge, and positional context. This enables the model to reflect the complexity of TCR–peptide recognition, including structural variability, latent interactions, and sequence similarity among TCRs targeting the same peptide.

Unlike traditional methods that use one-hot encoding or dimension reduction embedding [30, 40], and models such as ERGO-II, which applies autoencoding without accounting for positional information, or ATM-TCR, which employs a Transformer model without incorporating VJ gene and HLA type information, PepTCR-Net integrates diverse biological features (VJ gene, HLA types, and sequence similarity networks) within a modular design. This flexibility allows users to independently generate TCR embeddings or adapt the pipeline to new data. Importantly, our prediction model is based on the data from lab-verified TCR-antigen peptide recognitions, which differs from many existing methods that rely on artificially synthesized negative examples to create non-binding pair datasets.

Given the complexity of the adaptive immune system and the diversity of TCRs, our training data cannot comprehensively represent the entire space of possible TCR–peptide recognition. Nonetheless, we demonstrated that even when only TCR sequences are available, PE alone can yield strong predictive accuracy (Fig. 4A). Adding NE, which integrates ED-based TCR representations and TCR similarity networks substantially improves the performance (Figs. 3A, 4A and Supplementary Fig. 6). Though, as expected, the prediction performance decreases as the number of clusters increases, PepTCR-Net maintains high stability, especially when NE is included in the prediction model.

Our prediction model is based on BFNN, which provides probabilistic outputs rather than single-point predictions. This is especially valuable in immunology, where ambiguous recognition patterns and potential cross-reactivity are common. As demonstrated in Supplementary Fig. 4, BFNN can flag predictions with low confidence or potential multiple peptides, enhancing interpretability.

To ensure generalizability, we validated PepTCR-Net across ID and out-of-distribution. To mitigate potential biases arising from a class imbalance in our dataset, we applied a group-based random split of the entire dataset by peptide classes into training, validation, and testing sets. During training, we used the class weight technique [41, 42] during training to address the overrepresentation of certain peptides, ensuring the model paid attention to underrepresented (minority) classes. Additionally, we employed early stopping based on validation performance which helped prevent overfitting.

We also demonstrated a real-world application of our framework in predicting whether specific TCRs can recognize antigen peptides of interest, even without prior knowledge of a known TCR–peptide relationship. This enables our method to serve as a *de novo* TCR-antigen prediction tool for infectious diseases. Furthermore, it could be used as a diagnostic tool to identify patients with strong or weak immune responses to infection or vaccination, or as a biomarker for cancer immunotherapy responses based on predicted TCR–peptide recognition.

In addition, researchers can use our tools to prioritize TCRs that are likely to recognize specific antigen peptides, reducing the number of candidates for experimental validation. This targeted approach may lower laboratory costs and accelerate discovery. We have made our source code and pre-trained models publicly available, enabling users with basic programming skills to generate different types of TCR representations based on their specific needs. For prediction, users can utilize our model to determine whether their TCRs recognize any of the antigen peptides in our training dataset, as demonstrated in the Application to Real-World Data section. Users with their own datasets can train the model using our codebase, allowing them to tailor the framework to their specific research needs.

The proposed framework has several limitations: (i) Users must specify a list of antigen peptides of interest. (ii) Like most deep learning models, PepTCR-Net requires a large number of validated TCR–peptide pairs for model training. Fortunately, the rapid growth of large-scale sequencing datasets and curated public repositories (e.g. IEDB, VDJdb) will alleviate this challenge. (iii) As a classification model, PepTCR-Net may not detect TCRs that can recognize multiple peptides or none. While the BFNN partially addresses this limitation by introducing uncertainty estimates, further improvements are needed. Future extensions using probabilistic or multi-label learning approaches could better quantify prediction confidence or multi-label learning frameworks. (iv) Being sequence-based, the model may misclassify TCRs with subtle amino acid differences that lead to divergent specificities. However, ongoing advances in sequence representation techniques are expected to improve discrimination accuracy. Finally, while the framework is modular and adaptable, applying it to different immune contexts (e.g. autoimmunity or vaccine response) may require domain-specific adjustments. As more diverse datasets become available, retraining or fine-tuning the model for specific applications will help improve its utility across immunological and translational applications.

**Key Points**

- Unlike existing methods that focus on binary prediction tasks, we develop a multi-class antigen-specific T-cell receptor (TCR) prediction framework, enhancing the generalization and usability of TCR prediction tools.
- The model incorporates diverse biological data types (TCR Sequence, HLA Type, and VJ Gene information), addressing a gap in current methods.
- The framework was trained on large, publicly available datasets (IEDB, VDJdb, McPAS) and validated both internally and externally. Additionally, its applicability was demonstrated on a real-world dataset, showcasing the potential for broad application in immune response and disease research.
- These contributions highlight the novelty and practicality of the PepTCR-Net framework in advancing computational immunology.

## Supplementary Material

SupplementaryFigures_bbaf351

SupplementaryDocument_v3_bbaf351

## Data Availability

The data underlying this article are available in Zenodo at https://zenodo.org/records/14194728. The datasets were derived from sources in the public domain: https://www.iedb.org, https://vdjdb.cdr3.net/, https://friedmanlab.weizmann.ac.il/McPAS-TCR/, https://clients.adaptivebiotech.com/pub/covid-2020, and gateway.ireceptor.org. The codes are also available at https://doi.org/10.6084/m9.figshare.27874143.v2

## References

[1] Riley TP, Hellman LM, Gee MH. et al. T cell receptor cross-reactivity expanded by dramatic peptide-MHC adaptability. *Nat Chem Biol* 2018;14:934–42. 30224695 10.1038/s41589-018-0130-4 PMC6371774

[2] Yang H, Cham J, Fan Z. et al. NAIR: network analysis of immune repertoire. Front Immunol. 2023;14:1181825. 10.3389/fimmu.2023.1181825 PMC10443597 37614227

[3] Montemurro A, Schuster V, Povlsen HR. et al. NetTCR-2.0 enables accurate prediction of TCR–peptide binding by using paired TCRα and β sequence data. *Commun Biol* 2021;4:1060. Available from: https://www.nature.com/articles/s42003-021-02610-3 34508155 10.1038/s42003-021-02610-3 PMC8433451

[4] Henikoff S, Henikoff JG. Amino acid substitution matrices from protein blocks. *Proc Natl Acad Sci U S A* 1992;89:10915–9. 1438297 10.1073/pnas.89.22.10915 PMC50453

[5] Moris P, De Pauw J, Postovskaya A. et al. Current challenges for unseen-epitope TCR interaction prediction and a new perspective derived from image classification. *Brief Bioinform* 2021;22:bbaa318. Available from: 10.1093/bib/bbaa318/6042663 33346826 PMC8294552

[6] Zhang Z, Xiong D, Wang X. et al. Mapping the functional landscape of T cell receptor repertoires by single-T cell transcriptomics. *Nat Methods* 2021;18:92–9. Available from: https://www.nature.com/articles/s41592-020-01020-3 33408405 10.1038/s41592-020-01020-3 PMC7799492

[7] Atchley WR, Zhao J, Fernandes AD. et al. Solving the protein sequence metric problem. *Proc Natl Acad Sci U S A* 2005;102:6395–400. Available from: 10.1073/pnas.0408677102 15851683 PMC1088356

[8] Cai M, Bang S, Zhang P. et al. ATM-TCR: TCR-epitope binding affinity prediction using a multi-head self-attention model. *Front Immunol* 2022;13:893247. Available from: 10.3389/fimmu.2022.893247/full 35874725 PMC9299376

[9] Dahouda MK, Joe I. A deep-learned embedding technique for categorical features encoding. *IEEE Access* 2021;9:114381–91. Available from: https://ieeexplore.ieee.org/document/9512057/

[10] Vaswani A, Shazeer N, Parmar N. et al. Attention is all you need Proceedings of the 31st International Conference on Neural Information Processing Systems. 2017, Curran Associates Inc. pp. 6000–10.

[11] Bonsor DA, Sundberg EJ. Dissecting protein−protein interactions using directed evolution. *Biochemistry.* 2011;50:2394–402. Available from: 10.1021/bi102019c 21332192

[12] Sutskever I, Vinyals O, Le QV. Sequence to sequence learning with neural networks [internet] arXiv. Proceedings of the 28th International Conference on Neural Information Processing Systems - Volume 2, 2014, Montreal, Canada, Series NIPS'14, pp. 3104–12.

[13] Cho K, Van Merrienboer B, Gulcehre C. et al. Learning phrase representations using RNN encoder–decoder for statistical machine translation. In: Moschitti A, Pang B, Daelemans W. (eds), Proceedings of the 2014 Conference on Empirical Methods in Natural Language Processing (EMNLP) [Internet], pp. 1724–34. Doha, Qatar: Association for Computational Linguistics, 2014.

[14] Khemani B, Patil S, Kotecha K. et al. A review of graph neural networks: concepts, architectures, techniques, challenges, datasets, applications, and future directions. *J Big Data* 2024;11:18. Available from: 10.1186/s40537-023-00876-4

[15] Springer I, Tickotsky N, Louzoun Y. Contribution of T cell receptor alpha and Beta CDR3, MHC typing, V and J genes to peptide binding prediction. *Front Immunol* 2021;12:664514. Available from: 10.3389/fimmu.2021.664514/full 33981311 PMC8107833

[16] Gao Y, Gao Y, Fan Y. et al. Pan-peptide meta learning for T-cell receptor–antigen binding recognition. *Nat Mach Intell* 2023;5:236–49. Available from: https://www.nature.com/articles/s42256-023-00619-3

[17] Abdullah AA, Hassan MM, Mustafa YT. A review on Bayesian deep learning in healthcare: applications and challenges. *IEEE Access* 2022;10:36538–62. Available from: https://ieeexplore.ieee.org/document/9745083/

[18] Dürr O, Sick B, Murina E. In: Michaels M, Trimpe M, Zubarev A. et al. (eds), Probabilistic Deep Learning: With Python, Keras, and TensorFlow Probability. Shelter Island, NY: Manning, 2020. 274.

[19] Masegosa AR, Cabañas R, Langseth H. et al. Probabilistic models with deep neural networks. Entropy (Basel). 2021;23:117. 10.3390/e23010117 PMC7831091 33477544

[20] Vita R, Mahajan S, Overton JA. et al. The immune epitope database (IEDB): 2018 update. *Nucleic Acids Res* 2019;47:D339–43. Available from: https://academic.oup.com/nar/article/47/D1/D339/5144151 30357391 10.1093/nar/gky1006 PMC6324067

[21] Shugay M, Bagaev DV, Zvyagin IV. et al. VDJdb: a curated database of T-cell receptor sequences with known antigen specificity. *Nucleic Acids Res* 2018;46:D419–27. Available from: http://academic.oup.com/nar/article/46/D1/D419/4101254 28977646 10.1093/nar/gkx760 PMC5753233

[22] Tickotsky N, Sagiv T, Prilusky J. et al. McPAS-TCR: a manually curated catalogue of pathology-associated T cell receptor sequences Wren J, editor. *Bioinformatics.* 2017;33:2924–9. Available from: https://academic.oup.com/bioinformatics/article/33/18/2924/3803440 28481982 10.1093/bioinformatics/btx286

[23] Nolan S, Vignali M, Klinger M. et al. A large-scale database of T-cell receptor beta (TCRβ) sequences and binding associations from natural and synthetic exposure to SARS-CoV-2. Frontiers in Immunology, Volume 16, 2025. 10.3389/fimmu.2025.1488851 PMC11873104 40034696

[24] Glanville J, Huang H, Nau A. et al. Identifying specificity groups in the T cell receptor repertoire. *Nature.* 2017;547:94–8. Available from: https://www.nature.com/articles/nature22976 28636589 10.1038/nature22976 PMC5794212

[25] Pogorelyy MV, Minervina AA, Touzel MP. et al. Precise tracking of vaccine-responding T cell clones reveals convergent and personalized response in identical twins. *Proc Natl Acad Sci U S A* 2018;115:12704–9. Available from: 10.1073/pnas.1809642115 30459272 PMC6294963

[26] Rosenberg SA, Restifo NP, Yang JC. et al. Adoptive cell transfer: a clinical path to effective cancer immunotherapy. *Nat Rev Cancer* 2008;8:299–308. 18354418 10.1038/nrc2355 PMC2553205

[27] Zhou L, Zeng Z, Egloff AM. et al. Checkpoint blockade-induced CD8+ T cell differentiation in head and neck cancer responders. *J Immunother Cancer* 2022;10:e004034. Available from: 10.1136/jitc-2021-004034 35058328 PMC8772459

[28] Jurtz V, Paul S, Andreatta M. et al. NetMHCpan-4.0: improved peptide–MHC class I interaction predictions integrating eluted ligand and peptide binding affinity data. *The Journal of Immunology* 2017;199:3360–8. Available from: https://academic.oup.com/jimmunol/article/199/9/3360/7977122 28978689 10.4049/jimmunol.1700893 PMC5679736

[29] Hamilton WL, Ying R, Leskovec J. Inductive representation learning on large graphs, Publisher Curran Associates Inc., Proceedings of the 31st International Conference on Neural Information Processing Systems, Series NIPS'17, pp. 1025–35.

[30] Rodríguez P, Bautista MA, Gonzàlez J. et al. Beyond one-hot encoding: lower dimensional target embedding. *Image and Vision Computing* 2018;75:21–31. Available from: https://linkinghub.elsevier.com/retrieve/pii/S0262885618300623

[31] Santiago C, Barata C, Sasdelli M. et al. LOW: Training deep neural networks by learning optimal sample weights. Pattern Recognition, 2021;110:107585. 10.1016/j.patcog.2020.107585

[32] McInnes L, Healy J, Melville J. UMAP: uniform manifold approximation and projection for dimension reduction. Journal of Open Source Software, 3:861. 10.21105/joss.00861

[33] Rand WM . Objective criteria for the evaluation of clustering methods. *J Am Stat Assoc* 1971;66:846–50. Available from: 10.1080/01621459.1971.10482356

[34] Snyder TM, Gittelman RM, Klinger M. et al. Magnitude and Dynamics of the T-Cell Response to SARS-CoV-2 Infection at both Individual and Population Levels. Frontiers in Immunology, 2020;15:2025. 10.3389/fimmu.2024.1488860 PMC11747429 39840037

[35] Wang X, Zhang J, Liu M. et al. Nonconserved epitopes dominate reverse preexisting T cell immunity in COVID-19 convalescents. *Sig Transduct Target Ther* 2024;9:160. Available from: https://www.nature.com/articles/s41392-024-01876-3 10.1038/s41392-024-01876-3 PMC11169541 38866784

[36] Saini SK, Hersby DS, Tamhane T. et al. SARS-CoV-2 genome-wide T cell epitope mapping reveals immunodominance and substantial CD8^+^ T cell activation in COVID-19 patients. *Sci Immunol* 2021;6:eabf7550. Available from: 10.1126/sciimmunol.abf7550 33853928 PMC8139428

[37] Schultheiß C, Paschold L, Simnica D. et al. Next-generation sequencing of T and B cell receptor repertoires from COVID-19 patients showed signatures associated with severity of disease. *Immunity.* 2020;53:442–455.e4. 32668194 10.1016/j.immuni.2020.06.024 PMC7324317

[38] Pedregosa, Fabian, Varoquaux. et al. Scikit-learn, Journal of Machine Learning Research, 2011;12:2825–30.

[39] Mahesh B . Machine learning algorithms*—*a review. *IJSR.* 2020;9:381–6. Available from: https://www.ijsr.net/archive/v9i1/ART20203995.pdf

[40] Fischer DS, Wu Y, Schubert B. et al. Predicting antigen specificity of single T cells based on TCR CDR 3 regions. *Mol Syst Biol* 2020;16:e9416. Available from: 10.15252/msb.20199416 32779888 PMC7418512

[41] Zhu M, Xia J, Jin X. et al. Class weights random Forest algorithm for processing class imbalanced medical data. *IEEE Access.* 2018;6:4641–52. Available from: http://ieeexplore.ieee.org/document/8246503/

[42] Song B, Li S, Sunny S. et al. Classification of imbalanced oral cancer image data from high-risk population. *J Biomed Opt* 2021;26:105001. 10.1117/1.JBO.26.10.105001 PMC8536945 34689442

