# Supplementary material for: PepTCR-Net: prediction of multi-class antigen peptides by T-cell receptor sequences with deep learning
Source: Brief Bioinform. 2025 Jul 24;26(4):bbaf351. doi: 10.1093/bib/bbaf351 (PMC12286776; doi:10.1093/bib/bbaf351)
Supplement: SupplementaryFigures_bbaf351 [file supplementaryfigures_bbaf351.pdf]

Supplementary Figure 1

A

Single aa	Token Index	Polarity	Secondary Structure	Molecular Volume	Codon Diversity	Electro-static Charge	Average
A	1	-0.591	-1.302	-0.733	1.57	-0.146	-1.202
C	2	-1.343	0.465	-0.862	-1.02	-0.255	-3.015
D	3	1.05	0.302	-3.656	-0.259	-3.242	-5.805
E	4	1.357	-1.453	1.477	0.113	-0.837	0.657
F	5	-1.006	-0.59	1.891	-0.397	0.412	0.31
G	6	-0.384	1.652	1.33	1.045	2.064	5.707
H	7	0.336	-0.417	-1.673	-1.474	-0.078	-3.306
I	8	-1.239	-0.547	2.131	0.393	0.816	1.554
K	9	1.831	-0.561	0.533	-0.277	1.648	3.174
L	10	-1.019	-0.987	-1.505	1.266	-0.912	-3.157
M	11	-0.663	-1.524	2.219	-1.005	1.212	0.239
N	12	0.945	0.828	1.299	-0.169	0.933	3.836
P	13	0.189	2.081	-1.628	0.421	-1.392	-0.329
Q	14	0.931	-0.179	-3.005	-0.503	-1.853	-4.609
R	15	1.538	-0.055	1.502	0.44	2.897	6.322
S	16	-0.228	1.399	-4.76	0.67	-2.647	-5.566
T	17	-0.032	0.326	2.213	0.908	1.313	4.728
V	18	-1.337	-0.279	-0.544	1.242	-1.262	-2.18
W	19	-0.595	0.009	0.672	-2.128	-0.184	-2.226
Y	20	0.26	0.83	3.097	-0.838	1.512	4.861

B

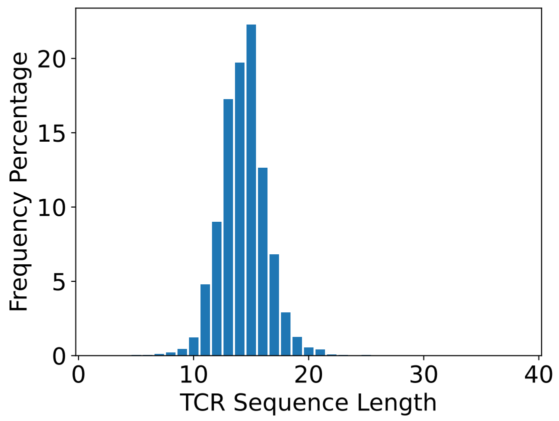

C

Tokenization

**Sequence**	AGVKITNSYEQYF
**Token**	A,G,V,K,I,T,N,S,Y,E,Q,Y,F
**Token index**	1,6,18,9,8,17,12,16,20,4,14,20,5

Embedding Lookup and Zero Padding

Token	Token Index	Vectorized TCR as final output
A	1	[-0.591, -1.302, -0.733, 1.57 , -0.146, -1.202]
G	6	[-0.384, 1.652, 1.33 , 1.045, 2.064, 5.707]
V	18	[-1.337, -0.279, -0.544, 1.242, -1.262, -2.18]
K	9	[1.831, -0.561, 0.533, -0.277, 1.648, 3.174]
I	8	[-1.239, -0.547, 2.131, 0.393, 0.816, 1.554]
T	17	[-0.032, 0.326, 2.213, 0.908, 1.313, 4.728]
N	12	[0.945, 0.828, 1.299, -0.169, 0.933, 3.836]
S	16	[-0.228, 1.399, -4.76 , 0.67 , -2.647, -5.566]
Y	20	[0.26 , 0.83 , 3.097, -0.838, 1.512, 4.861]
E	4	[1.357, -1.453, 1.477, 0.113, -0.837, 0.657]
Q	14	[0.931, -0.179, -3.005, -0.503, -1.853, -4.609]
Y	20	[0.26 , 0.83 , 3.097, -0.838, 1.512, 4.861]
F	5	[-1.006, -0.59 , 1.891, -0.397, 0.412, 0.31]
	0	[0. , 0. , 0. , 0. , 0. , 0.] *x 25*

Supplementary Figure 2

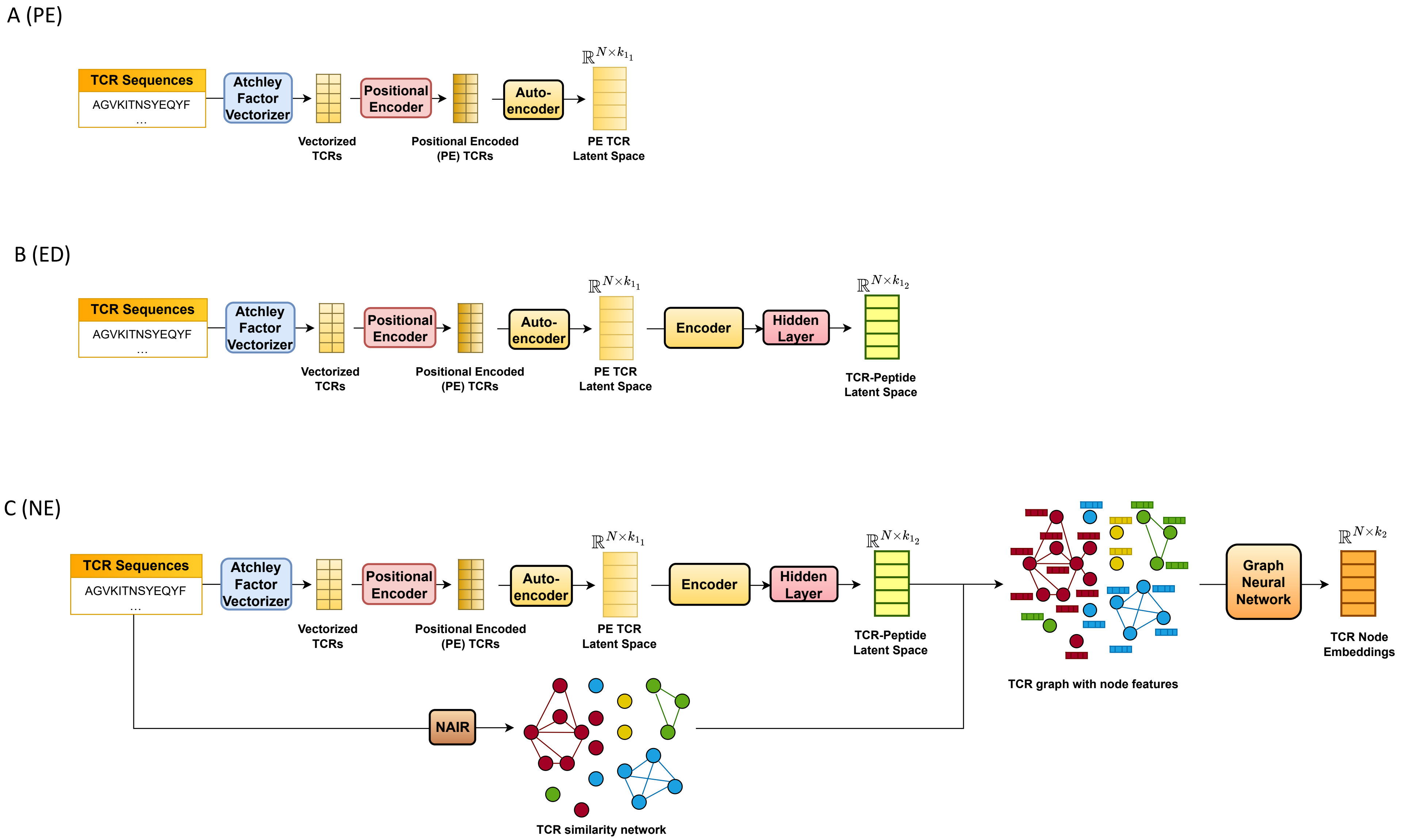

Supplementary Figure 3

A

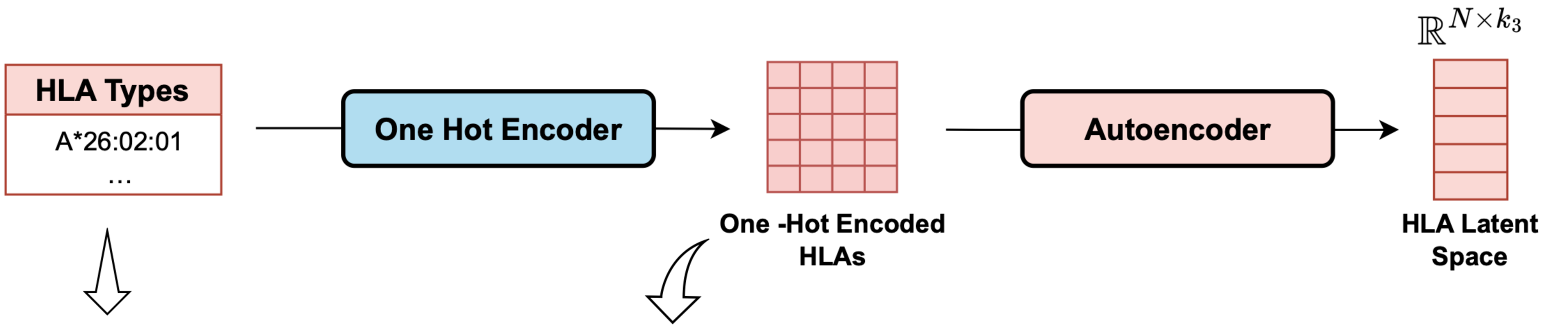

B

	MHC-A
1	HLA-A*03:01
2	HLA-A*02:01
3	HLA-A*11:01
4	HLA-B*08:01

C

	MHC-A_HLA-A*02:01	MHC-A_HLA-A*03:01	MHC-A_HLA-A*11:01	MHC-A_HLA-B*08:01
0	**1.0**	0.0	0.0	0.0
1	0.0	0.0	0.0	0.0
2	0.0	0.0	0.0	**1.0**
...	...	...	...	...
53248	0.0	**1.0**	0.0	0.0
53249	0.0	0.0	0.0	0.0

Supplementary Figure 4

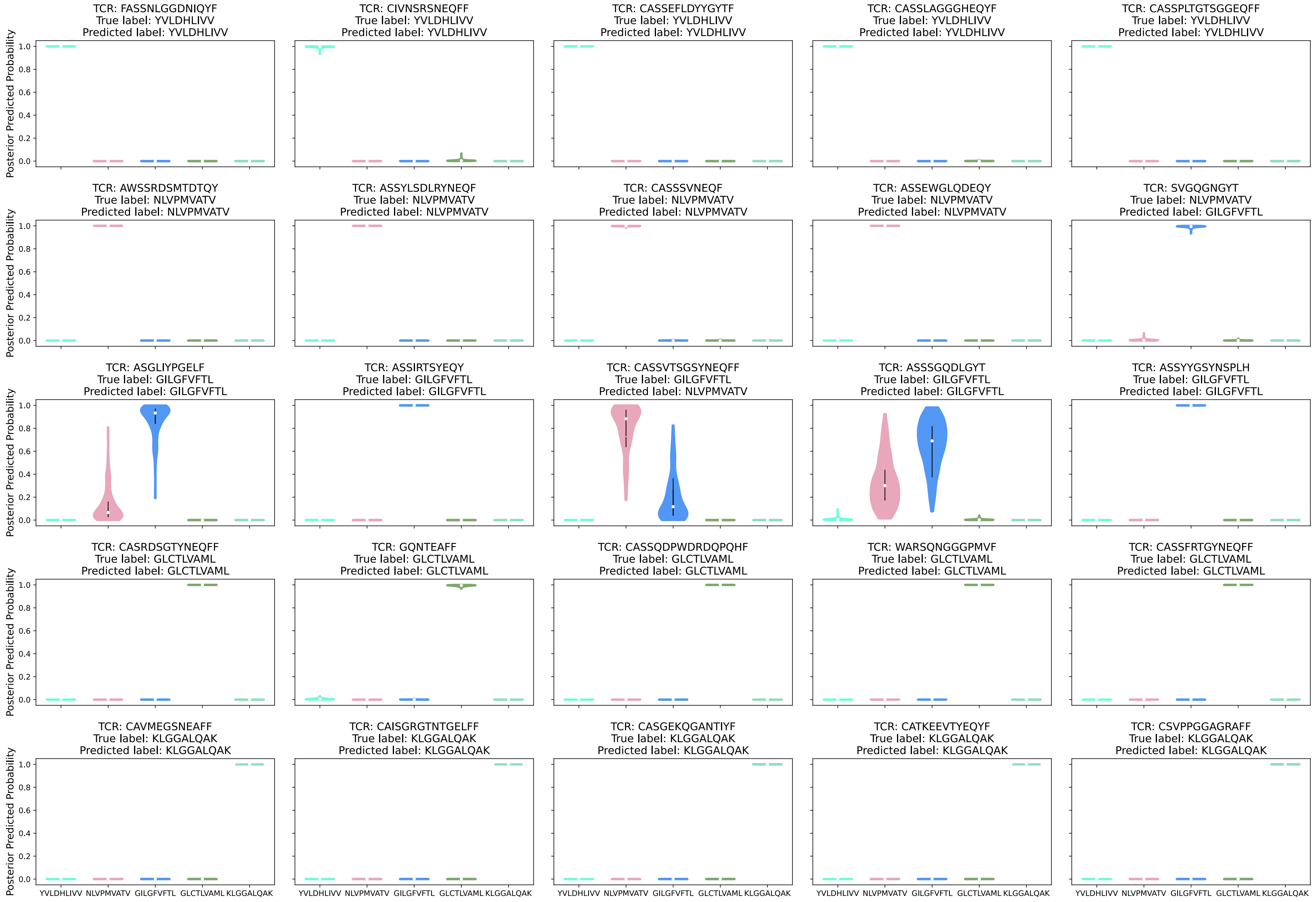

Supplementary Figure 5

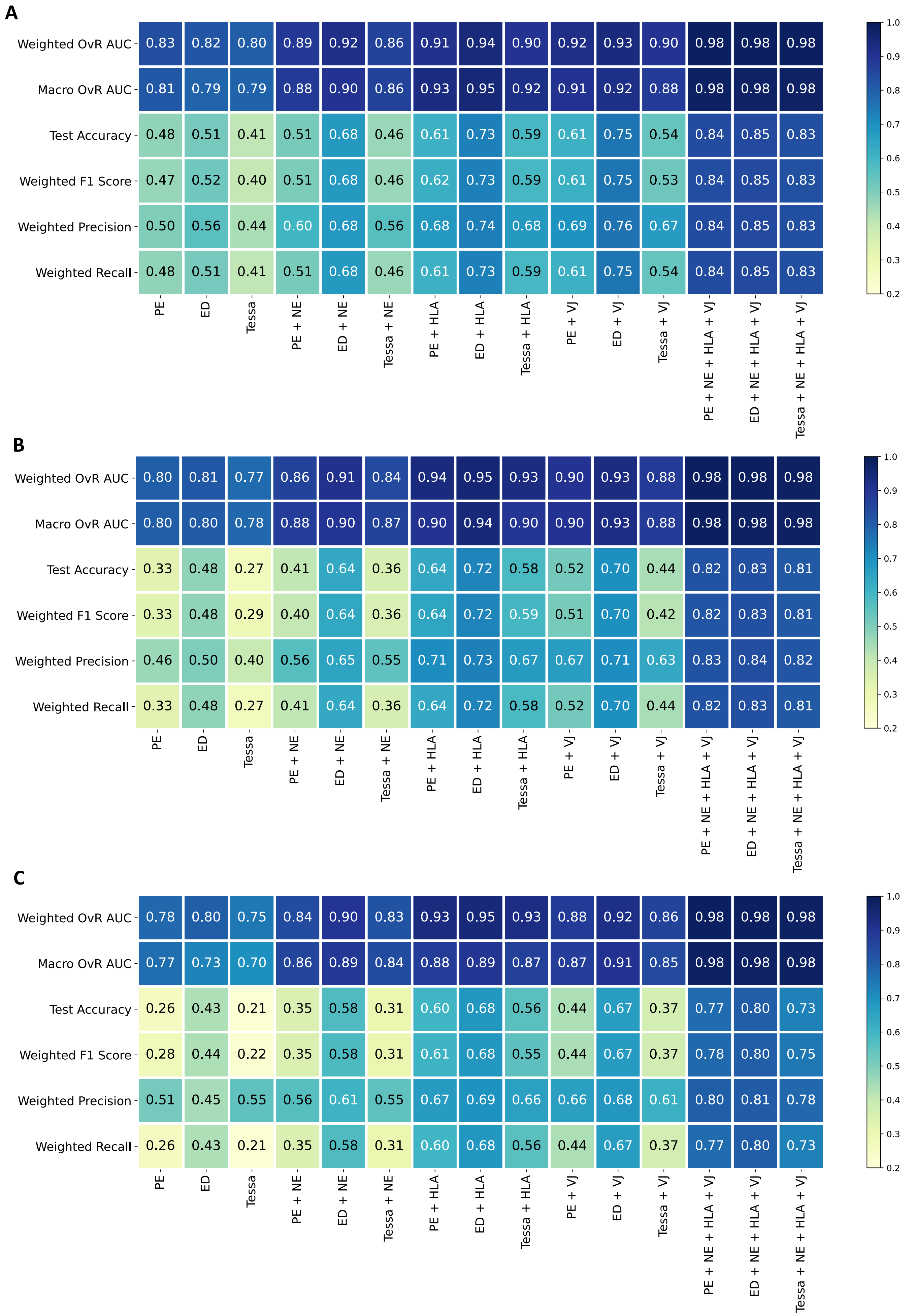

Supplementary Figure 6

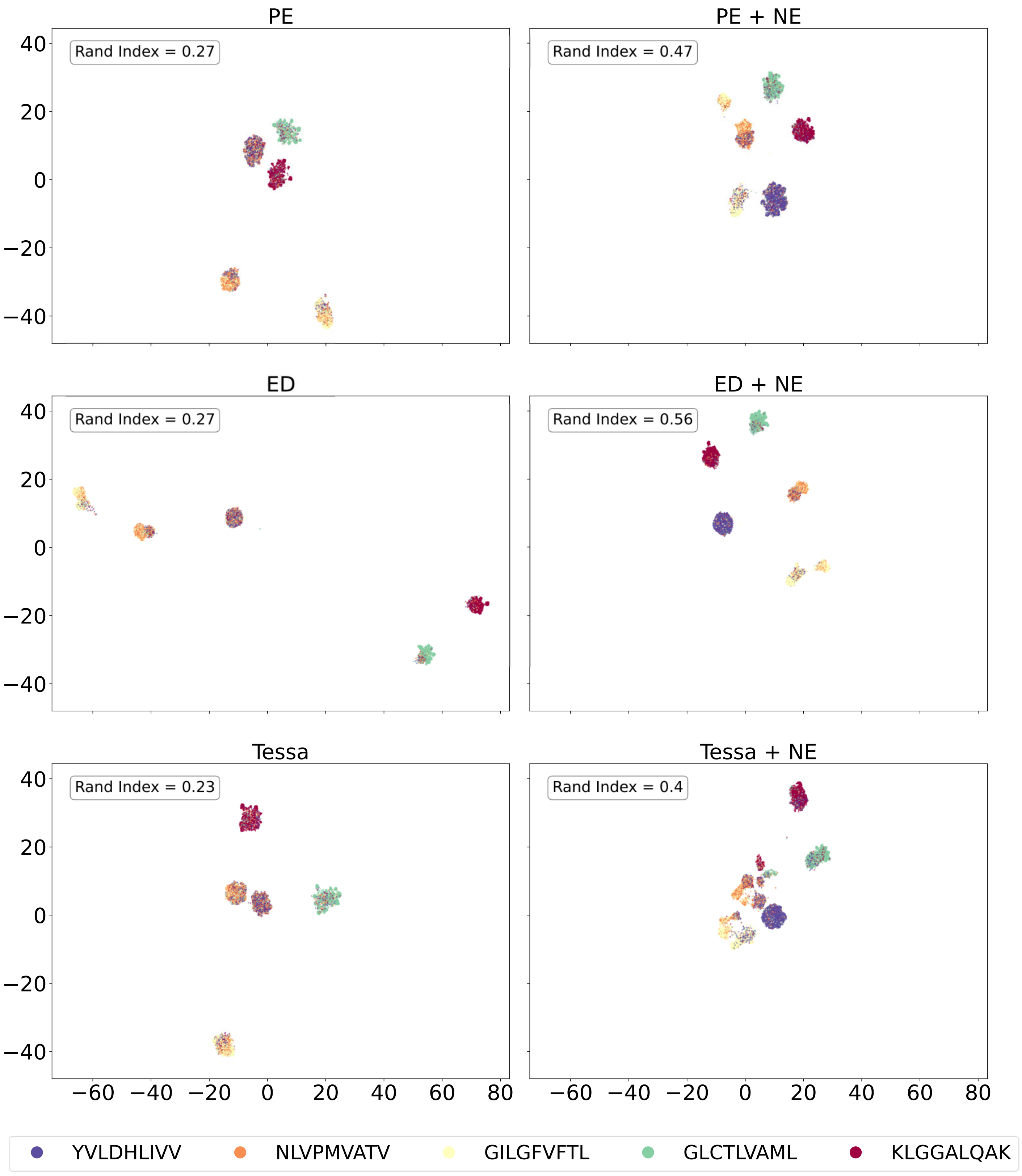

Supplementary Figure 7

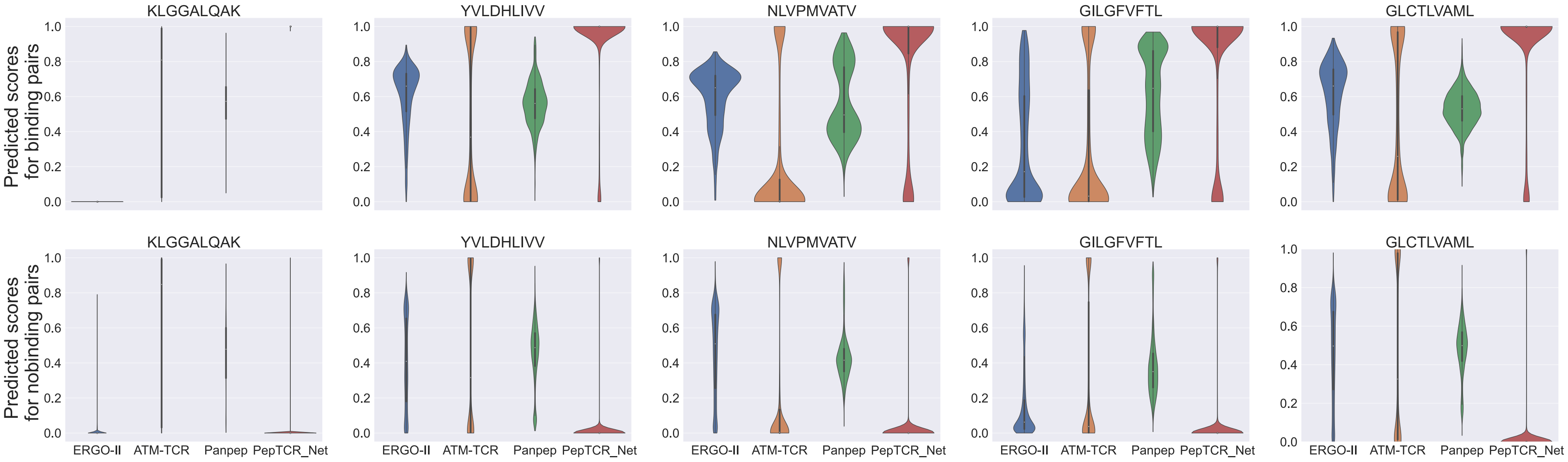

Supplementary Figure 8

Random Forest

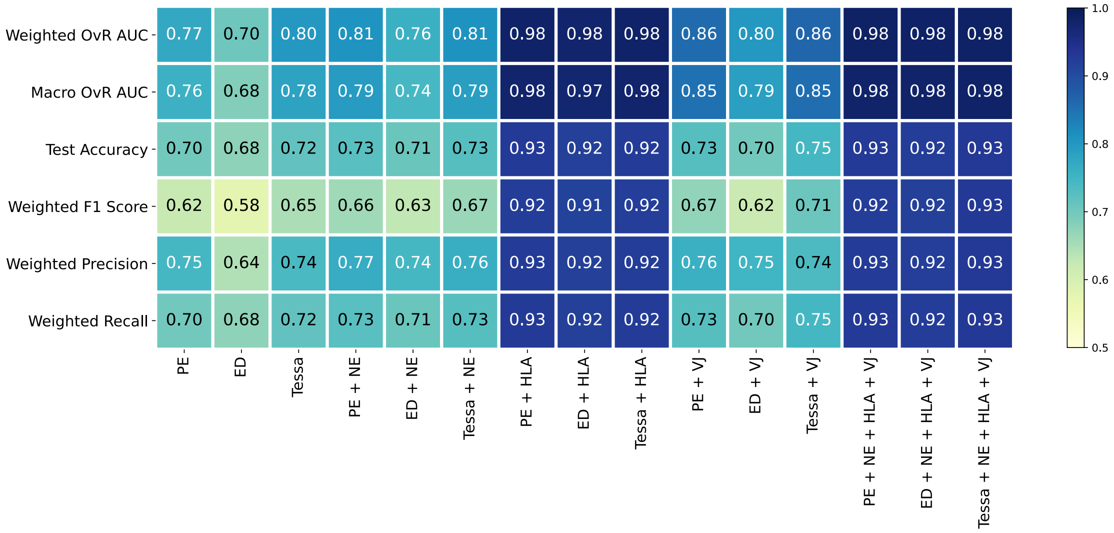

SVM

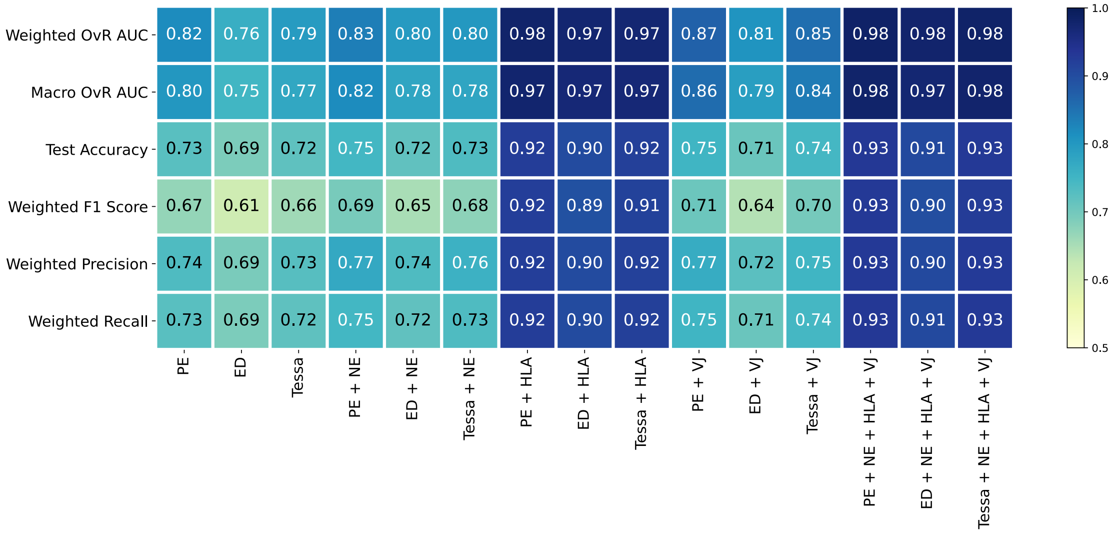

XGBoost

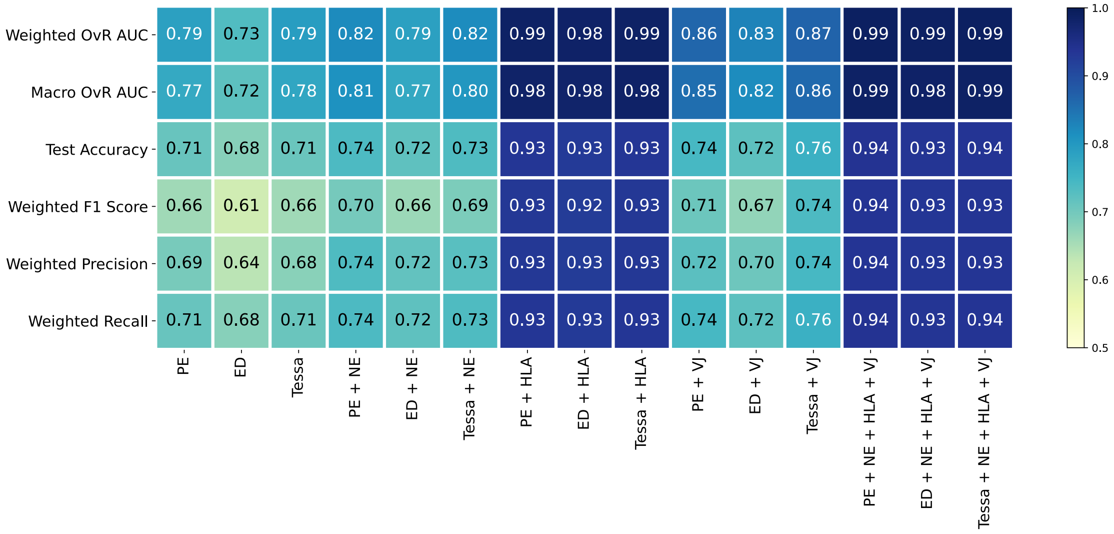

Supplementary Figure Legends

Supplementary Figure 1: Detailed information on encoding TCR sequences using Atchley factors.

(A) A lookup table of the 20 single amino acids for the five Atchley factors. (B) The bar chart represents the distribution of sequence lengths in the ED train dataset. The x-axis indicates the TCR sequence length, while the y-axis depicts the frequency percentage for each length. **(C) Atchley factor vectorization task - tokenization step.** This graph depicts the tokenization step in the Atchley factor vectorization task. It begins with a specific TCR sequence input. This sequence is subsequently split into individual letters, also known as single amino acids. A lookup table is then used to map the corresponding token index for each single amino acid. **(D) Atchley factor vectorization task - embedding lookup and zero padding step.** The token index, which is the output of the tokenization step, is used as input for the embedding lookup step. In this step, the lookup table is used again to map each token index to its corresponding Atchley factor vector. If the input sequence's length is less than the global maximum length, zero padding is applied. The final output is a matrix with dimensions [global maximum length X 6].

Supplementary Figure 2: Details on generating embeddings from TCR Sequences

(A) Positional Encoder (PE) (B) Encoder Decoder (ED) (C) Node Embedding (NE)

Supplementary Figure 3: Details on generating embeddings from categorical features (HLA Types and VJ Genes).

(A) Detailed steps for generating embeddings for HLA types and VJ genes. This module accepts VJ genes and HLA types as input, then employs a One Hot Encoder to generate one-hot encoded features. To minimize noise and reduce dimensionality, an autoencoder is used. The

final layer of the encoder block in the model is extracted to generate the HLA and VJ gene latent spaces. **(B) Example of HLA types in its categorical form. (C) Example of HLA embeddings after applying one-hot encoder.**

Supplementary Figure 4: Violin plots of the posterior prediction probability based on the ID dataset. Each panel plots the Posterior Prediction Probability of the top five most frequent peptides based on the Bayesian Feed Forward Model with ED+NE+HLA+VJ as the inputs. Each row represents one of the top 5 peptides (i.e., the true label). For each true peptide, we randomly selected five TCRs (representing by column), then plotted the distribution of the posterior prediction probability (based on 200 sampling) of each TCR for each predicted peptide (x-axis). The predicted label is based on the highest prediction probability within each panel. For example, the first panel in the first row one, for TCR CASCSLTGSGETLYF, the true peptide is YVLDHLIVV which is also the predicted peptide.

Supplementary Figure 5: Performance evaluation of PepTCR-Net on ID dataset across different number of classes. Heatmap depicting the prediction performance of the top ten **(A)**, fifteen **(B)** and twenty **(C)** most frequent peptides based on the Bayesian Feed Forward (BFF) model with different prediction inputs. The prediction performance was evaluated based on six metrics (by row): weighted One-vs-Rest (OvR) AUC, macro OvR AUC, test accuracy, weighted F1 score, weighted precision, and weighted recall. This evaluation was carried out for fifteen variations of inputs for the prediction model (by column): encoded TCR sequences alone (PE, ED, and Tessa); encoded TCR sequences combined with Node Embeddings (NE), i.e., PE+NE, ED+NE and Tessa+NE; encoded TCR sequences combined with HLA types, i.e., PE+HLA, ED+HLA and Tessa+HLA; encoded TCR sequences combined with VJ genes, i.e., PE+VJ, ED+

VJ and Tessa+ VJ; encoded TCR sequences combined with NE, HLA types, and VJ genes simultaneously, i.e., PE+NE+HLA+VJ, ED+NE+HLA+VJ and Tessa+NE+HLA+VJ.

Supplementary Figure 6: UMAP Plot of the embeddings of TCR sequences based on the ID dataset. (A) Encoder Decoder (ED) embedding model (B) ED plus Node Embedding (NE) (C) Positional Encoder (PE) (D) PE plus NE (E) Tessa (F) Tessa plus NE. For each figure, we applied the supervised UMAP using the predicted label information based on the corresponding embedding approach. The UMAP was color-coded based on the five most frequent peptides. The Rand Index (RI) was used to assess the consistency of clustering with the true peptide groups.

Supplementary Figure 7: Comparison results of the five most frequent peptides of the ID dataset Each panel plots the distribution of the predicted score of the binding pair (top row) and the nonbinding pair (bottom row) for each method (x-axis) for each peptide (column). The proposed PepTCR_Net is based on the Bayesian Feed Forward Model with ED+NE+HLA+VJ as the inputs.

Supplementary Figure 8: Performance evaluation of traditional machine learning approaches on OOD dataset. Heatmap depicting the prediction performance of the four peptide classes based on the three machine learning models with different prediction inputs. The prediction performance was evaluated based on six metrics (by row): weighted One-vs-Rest (OvR) AUC, macro OvR AUC, test accuracy, weighted F1 score, weighted precision, and weighted recall. This evaluation was carried out for fifteen variations of inputs for the prediction model (by column): encoded TCR sequences alone (PE, ED, and Tessa); encoded TCR sequences combined with Node Embeddings (NE), i.e., PE+NE, ED+NE and Tessa+NE; encoded TCR sequences combined with HLA types, i.e., PE+HLA, ED+HLA and Tessa+HLA;

encoded TCR sequences combined with VJ genes, i.e., PE+VJ, ED+ VJ and Tessa+ VJ; encoded TCR sequences combined with NE, HLA types, and VJ genes simultaneously, i.e., PE+NE+HLA+VJ, ED+NE+HLA+VJ and Tessa+NE+HLA+VJ.
