# Supplementary material for: PepTCR-Net: prediction of multi-class antigen peptides by T-cell receptor sequences with deep learning
Source: Brief Bioinform. 2025 Jul 24;26(4):bbaf351. doi: 10.1093/bib/bbaf351 (PMC12286776; doi:10.1093/bib/bbaf351)
Supplement: SupplementaryDocument_v3_bbaf351 [file supplementarydocument_v3_bbaf351.docx]

**Supplementary Document**

**METHOD**

**Atchley Factor Vectorization Task**

Atchley Factor Vectorization includes several steps, with the details in the Supplementary Document and Supplementary Figure 1. We first break down an amino acid sequence into tokens, each representing a single amino acid (Supplementary Figure 1A). Then, we map each single amino acid to a vector from an embedding matrix based on the token index, where the Atchley factors are used as the embedding matrix (Supplementary Figure 1B). As a result, an amino acid sequence becomes a k x 6 matrix, where k represents the maximal length of the sequence (the maximum lengths of TCRs and peptides are 35 and 25, respectively (Supplementary Figure 1C)). For sequences less than k (the maximum length of TCR sequences), we add zeros to the end of the sequence so that the vector representations obtained from the matrix always have the same length.

**Prediction Model by Bayesian Feedforward Neural Networks**

Suppose $y=f\left( x,\theta\right)$ estimates the output y given the input x and the parameters $\theta$ (model’s weights and biases) to produce a predictive output. The parameters $\theta$ are estimated by using the Bayesian posterior distribution

$p\left( \theta| D \right) \sim p\left( D | \theta\right)p\left( \theta\right)$,

where the prior distribution $p\left( \theta\right)$ follows a standard normal distribution and $D=\left( X,Y \right)$is a given training data with X as the predictor inputs of the prediction model and Y as the corresponding outcomes. The BFNN is trained from the data using Bayesian inference (1) to infer the posterior distribution of the parameters. Then, with a new predictor input data (for the prediction model) $x*$, TCR sequences with or without HLA type and VJ genes, we can obtain the predictive distribution of the corresponding outcome $y*$with likelihood function $p\left( y* | x*,\theta\right)$ as below

$$p\left( y* | x*,D \right)=\int p\left( y* | x*,\theta\right)p\left( \theta| D \right)d\theta$$

Since the posterior $p\left( \theta| D \right)$ in neural networks is untractable over multiple layers of networks, we approximate it with variational distributions $q\left( \theta| \theta^{'} \right)$ of known functional forms such as Gaussian distributions. To archive this, we minmize the Kullback-Leibler divergence (2) cost function (variational free energy) between $p\left( \theta| D \right)$ and $q\left( \theta| \theta^{'} \right)$

$$\mathcal{F}\left( D, \theta^{'} \right)=KL(q\left( \theta| \theta^{'} \right)\left| p\left( \theta\right) \right)-E_{q\left( \theta| \theta^{'} \right)}log(D|\theta)$$

We can approximate $\mathcal{F}\left( D, \theta^{'} \right)$ by drawing samples $\theta$ from $q\left( \theta| \theta^{'} \right)$

$$\mathcal{F}\left( D, \theta^{'} \right)= \frac{1}{N}\sum_{i=1}^{N} [\log q\left( \theta^{i} | \theta^{'} \right)-\log p\left( \theta^{i} \right)-\log p(D|\theta^{i})]$$

The parameters are determined by iterations of forward and backward passes during neural network training. For further details on approximation functions and training methods, we refer readers to the paper (3). Our model is implemented using the TensorFlow Probability library (4), eliminating the need to code it from scratch.

We extract 200 samples from the predictive distribution $p\left( y* | x*,D \right)$to obtain the sampling probabilities of TCR-peptide recognition. The antigen peptide with the highest median sampling probabilities is identified as the predicted peptide that the predictor input TCR can recognize. Moreover, the dispersion of the predicted probabilities across the antigen peptide classes offers insights into prediction uncertainty. Since the known TCRs binding to each peptide varies greatly, this unbalanced data can lead to biased learning as the model may favor the more frequently observed class. To mitigate this, we employed the sample weight learning technique (5) to help the model learn to reduce the effects of dominant classes during training by adjusting the loss function. For optimal learning, we adopted a tailored learning strategy, leveraging grid search to fine-tune sample weights for each peptide. The reciprocal of each class’s cardinality to that of the largest was selected as the sample weights.

**Metrics of Prediction Performance**

We evaluated our prediction model performance using a set of complementary metrics. Test accuracy measures the overall percentage of correct predictions. Since our task involves multi-class peptide prediction with potentially imbalanced class distributions, we used weighted One-vs-Rest AUC (6), which assesses how well the model distinguishes each peptide class from all others while giving more weight to common classes; we also used macro One-vs-Rest AUC (6) which does the same but treats all classes equally, regardless of their frequency. Precision is calculated as the proportion of the predicted positive recognitions that were actually correct, while recall measures the proportion of the true positive recognitions the model was able to identify. Weighted F1 score is the harmonic mean of precision and recall while accounting for class imbalance.

**RESULT**

**Consistent Performance across Different Numbers of Classes**

To examine the impact of the number of classes, we conducted experiments in a multi-class classification setting with different numbers of classes, specifically five, ten, fifteen, and twenty, in the in-distribution setup. As expected in a multi-class setting, accuracy decreases as the number of classes increases (Figure 3D) due to a high risk of misclassification. Nevertheless, the ED model consistently outperforms Tessa across different class sizes, and including NE further improves performance. Not surprisingly, the full model, which includes the VJ gene and HLA type, achieves the best results. In addition, the weighted One-vs-Rest AUC for the top five antigen peptides demonstrates the robustness and stability of our proposed models regardless of the number of classes (Figure 3E). While all TCR embeddings (PE, ED, and Tessa) exhibit some sensitivity to increasing class numbers, shown by a gradual decline in AUCs, PE and ED consistently outperform Tessa. This pattern holds even when additional information, such as NE or NE, HLA type, and VJ genes, are included. Supplementary Figures 5A, 5B, and 5C present the prediction performance of the top ten, fifteen, and twenty most frequent peptides based on the ID data using different prediction inputs. Although, as expected, the prediction performance generally decreases with more classes, the models remain stable, especially when NE is added, and the full models are even more robust compared to the model solely with TCR sequences.

**Comparison of Different Feature Engineering Approaches**

As mentioned above, the NE provides a robust alternative to enhance the prediction performance in cases where the HLA type is unavailable and/or the VJ gene annotation is incomplete or missing. Here, we conduct experiments on the ID dataset for the five most common peptides to evaluate the impact of adding NE to the TCR embeddings engineered by PE, ED, and Tessa on clustering quality. We used supervised UMAP (7) for each set of embeddings using predicted label information to project the high-dimensional feature space into two dimensions. A scatterplot was then constructed and color-coded by the true peptide information, i.e., the top five most frequent peptides. We configured the algorithm with parameters, where the number of nearest neighbors equals to 50 and the minimum distance to control within the cluster is 0.9. Supplementary Figures 6A, 6B, and 6C show the clustering results of the TCR embeddings: PE, ED, and Tessa, respectively. Both ED and PE have a rand index (8) of 0.27, indicating a moderate clustering quality and better class separation than Tessa (rand index =0.23). Supplementary Figures 6D, 6E, and 6F display the TCR embeddings plus NE embeddings (PE + NE, ED + NE, and Tessa + NE, respectively). These figures show a more apparent cluster distinction than using the TCR embeddings alone. Specifically, ED+NE has the best clustering quality with a rand index of 0.56, followed by PE + NE at 0.47 and Tessa + NE at 0.4. Those results present that incorporating NE improves the clustering structure and, therefore, the prediction performance.

**Comparison with Existing Methods**

To the best of our knowledge, our method is the first approach to predict if a TCR can recognize a given antigen peptide by using experimental validated (wet lab) data without relying on artificially synthesized data, which is commonly employed in other TCR-Peptide methods. Therefore, we cannot make a direct comparison with existing methods, such as ERGO-II(9), ATM-TCR(10), and Panpep(11), using the standard binary prediction metrics (such as AUC, F-1, etc.). Due to not knowing the optimal thresholds of other models, we compared the distribution of the recognition prediction scores across the methods, where a higher score indicates a higher likelihood of prediction recognition. For each antigen peptide, we computed the predicted scores from each method and compared the distributions of predicted scores between true recognizing (label 1) and true non-recognizing (label 0) TCRs. To obtain the prediction scores from ATM-TCR and ERGO-II, we applied their pre-trained model from their respective GitHub repositories directly to our ID dataset. For the Panpep model, we used their default settings under the majority learning scheme. Since Panpep also requires additional fine-tuning before making predictions, we provided our validation dataset as the fine-tuning inputs.

Supplementary Figure 7 presents the distributions of the prediction scores of true recognizing (label 1) and true non-recognizing (label 0) TCRs across the top five most frequent peptides from the ID dataset. Our proposed PepTCR-Net utilized the BFNN model with ED+NE+HLA+VJ as the input, consistently outperforms other approaches, achieving the highest prediction scores for true recognizing TCRs and the lowest median prediction scores for true non-recognizing TCRs. Importantly, it is trained on lab-validated TCR-peptide interactions, avoiding the potential biases introduced by artificially synthesized negative pairs. Since such synthetic negatives can distort learning, model performance across methods may vary depending on peptide diversity, class balance, and thresholding strategies. Thus, while PepTCR-Net shows superior performance, comparisons should be interpreted within this methodological context. It is important to note that because those existing approaches used synthesized negative examples during training, their performance may be sensitive to the quantity and characteristics of such negatives. Moreover, differences in the number of peptides used during training and variability in threshold selection across models can also impact the comparative performance assessments. Therefore, while our findings suggest superior performance, they should be interpreted in the context of these methodological differences.

We further expanded to include comparisons with traditional machine learning models (Random Forest, Support Vector Machines, and XGBoost) (12) based on the MIRA dataset. As shown in Supplementary Figure 8 while the BFNN models achieve performance comparable to these alternatives across multiple performance metrics, their probabilistic nature offers added interpretability, enabling researchers to better understand uncertainty and model behavior—something standard classifiers cannot provide.

**References**

1. Chang DT. Bayesian Neural Networks: Essentials. 2021; Available from: https://arxiv.org/abs/2106.13594

2. Hershey JR, Olsen PA. Approximating the Kullback Leibler Divergence Between Gaussian Mixture Models. In: 2007 IEEE International Conference on Acoustics, Speech and Signal Processing - ICASSP ’07 [Internet]. Honolulu, HI: IEEE; 2007. p. IV-317-IV–320. Available from: https://ieeexplore.ieee.org/document/4218101/

3. Shridhar K, Laumann F, Liwicki M. A Comprehensive guide to Bayesian Convolutional Neural Network with Variational Inference [Internet]. arXiv; 2019. Available from: https://arxiv.org/abs/1901.02731

4. Abadi M, Agarwal A, Barham P, Brevdo E, Chen Z, Citro C, et al. TensorFlow: Large-Scale Machine Learning on Heterogeneous Distributed Systems [Internet]. arXiv; 2016. Available from: https://arxiv.org/abs/1603.04467

5. Gao T, Jojic V. SAMPLE IMPORTANCE IN TRAINING DEEP NEURAL NETWORKS. In. Available from: https://openreview.net/pdf?id=r1IRctqxg

6. Scikit-learn. ROC metrics - Scikit-learn [Internet]. Available from: https://scikit-learn.org/stable/modules/model_evaluation.html#roc-metrics

7. McInnes L, Healy J, Melville J. UMAP: Uniform Manifold Approximation and Projection for Dimension Reduction [Internet]. arXiv; 2018. Available from: https://arxiv.org/abs/1802.03426

8. Rand WM. Objective Criteria for the Evaluation of Clustering Methods. Journal of the American Statistical Association [Internet]. 1971 Dec 66(336):846–50. Available from: http://www.tandfonline.com/doi/abs/10.1080/01621459.1971.10482356

9. Springer I, Tickotsky N, Louzoun Y. Contribution of T Cell Receptor Alpha and Beta CDR3, MHC Typing, V and J Genes to Peptide Binding Prediction. Front Immunol [Internet]. 2021 Apr 26;12:664514. Available from: https://www.frontiersin.org/articles/10.3389/fimmu.2021.664514/full

10.Cai M, Bang S, Zhang P, Lee H. ATM-TCR: TCR-Epitope Binding Affinity Prediction Using a Multi-Head Self-Attention Model. Front Immunol [Internet]. 2022 Jul 6;13:893247. Available from: https://www.frontiersin.org/articles/10.3389/fimmu.2022.893247/full

11.Gao Y, Gao Y, Fan Y, Zhu C, Wei Z, Zhou C, et al. Pan-Peptide Meta Learning for T-cell receptor–antigen binding recognition. Nat Mach Intell [Internet]. 2023 Mar 6 [;5(3):236–49. Available from: https://www.nature.com/articles/s42256-023-00619-3

12.Mahesh B. Machine Learning Algorithms - A Review. IJSR [Internet]. 2020 Jan 5;9(1):381–6. Available from: https://www.ijsr.net/archive/v9i1/ART20203995.pdf

**Supplementary Table 1 Summary of the Datasets**

| **Datasets** | **Unique TCR** | **Unique Peptide** | **Unique Pair** | **Percentage Overlapping with SE Train Dataset** |
| --- | --- | --- | --- | --- |
| **SE Train Dataset** | 164,891 | 2,443 | 179,473 | 100% |
| **ID Prediction Dataset** | 76,445 | 1,601 | 84,804 | 71.12% |
| **OOD Prediction Dataset** | 15,201 | 4 | 15,221 | 4.53% |

**Supplementary Table 2: Top counts of HLA types for each peptide from ID dataset**

| ID data | | |
| --- | --- | --- |
| Peptide | MHC | count |
| GILGFVFTL | HLA-A*02:01 | 8881 |
|  | HLA-A*02 | 2205 |
|  | HLA-A2 | 788 |
|  | HLA-A*02:01:48 | 7 |
|  | HLA-A*02:04 | 1 |
| GLCTLVAML | HLA-A*02:01 | 11783 |
|  | HLA-A2 | 743 |
|  | HLA-A*02 | 285 |
|  | HLA-A*2:01 | 7 |
|  | HLA-A*02:16 | 1 |
| KLGGALQAK | HLA-A*03:01 | 25683 |
| NLVPMVATV | HLA-A*02:01 | 5846 |
|  | HLA-A*02 | 4155 |
|  | HLA-A2 | 281 |
|  | HLA-A*02:01:110 | 1 |
|  | HLA-A*02:01:59 | 1 |
| YVLDHLIVV | HLA-A*02:01 | 17012 |
|  | HLA-A*02 | 17 |
|  | HLA-A2 | 6 |
|  | HLA-A*01 | 3 |

| **Supplementary Table 3: Variance estimate of the performance metrics for Pep-TCR Net on ID Prediction Dataset** | | | | | | |
| --- | --- | --- | --- | --- | --- | --- |
|  | Weighted OvR AUC | Macro OvR AUC | Test Accuracy | Weighted F1 Score | Weighted Precision | Weighted Recall |
| PE | 3.33E-10 | 2.3E-09 | 2.24E-07 | 2.09E-07 | 2.54E-07 | 2.24E-07 |
| ED | 2.71E-09 | 2.57E-09 | 1.21E-07 | 1.13E-07 | 1.30E-07 | 1.21E-07 |
| Tessa | 3.33E-10 | 1.44E-09 | 2.68E-07 | 2.85E-07 | 3.23E-07 | 2.68E-07 |
| PE + NE | 1.28E-32 | 5.1E-32 | 1.24E-07 | 1.08E-07 | 1.03E-07 | 1.24E-07 |
| ED + NE | 5.1E-32 | 3.33E-10 | 1.10E-07 | 1.18E-07 | 1.31E-07 | 1.10E-07 |
| Tessa + NE | 3.33E-10 | 2.57E-09 | 1.24E-07 | 1.14E-07 | 1.06E-07 | 1.24E-07 |
| PE + HLA | 2.02E-09 | 6.44E-10 | 1.38E-07 | 1.52E-07 | 1.62E-07 | 1.38E-07 |
| ED + HLA | 2.34E-09 | 2.86E-09 | 1.72E-07 | 1.74E-07 | 1.67E-07 | 1.72E-07 |
| Tessa + HLA | 2.54E-09 | 2.17E-09 | 4.30E-07 | 4.83E-07 | 5.46E-07 | 4.30E-07 |
| PE + VJ | 9.31E-10 | 1.44E-09 | 5.41E-08 | 6.05E-08 | 6.40E-08 | 5.41E-08 |
| ED + VJ | 5.1E-32 | 1.44E-09 | 8.95E-08 | 9.45E-08 | 1.01E-07 | 8.95E-08 |
| Tessa + VJ | 3.33E-10 | 1.28E-32 | 7.43E-08 | 7.24E-08 | 6.36E-08 | 7.43E-08 |
| PE + HLA + NE + VJ | 9.31E-10 | 6.44E-10 | 6.38E-08 | 5.84E-08 | 6.55E-08 | 6.38E-08 |
| ED + HLA + NE + VJ | 3.33E-10 | 2.4E-09 | 2.86E-08 | 2.35E-08 | 2.75E-08 | 2.86E-08 |
| Tessa + HLA + NE + VJ | 2.3E-09 | 3.09E-09 | 4.81E-08 | 5.21E-08 | 5.33E-08 | 4.81E-08 |

**Supplementary Table 4: Top counts of HLA types for each peptide from OOD dataset**

| OOD | | | | |
| --- | --- | --- | --- | --- |
| Peptide | HLA.A | HLA.B | HLA.C | count |
| AYKTFPPTEPK,KTFPPTEPK | A*03:01:01 | B*08:01:01 | C*03:04:01 | 230 |
|  | A*02:01:01 | B*07:02:01 | C*07:01:01 | 114 |
|  | A*02:03:01 | B*39:01:01 | C*07:02:01 | 106 |
|  | A*11:01:01 | B*08:01:01 | C*07:01:01 | 88 |
|  | A*03:01:01 | B*07:02:01 | C*07:02:01 | 73 |
| HTTDPSFLGRY | A*01:01:01 | B*07:02:01 | C*07:01:01 | 2598 |
|  |  | B*08:01:01 | C*03:04:01 | 1239 |
|  |  | B*15:02:01 | C*01:02:01 | 913 |
|  |  | B*08:01:01 | C*07:01:01 | 900 |
|  |  | B*56:01:01 | C*01:02:01 | 880 |
| KAYNVTQAF | A*11:01:01 | B*08:01:01 | C*07:01:01 | 298 |
|  | A*32:01:01 | B*40:02:01 | C*12:02:02 | 292 |
|  | A*03:01:01 | B*07:02:01 | C*06:02:01 | 182 |
|  | A*24:02:01 | B*40:02:01 | C*01:02:01 | 80 |
|  | A*01:01:01 | B*49:01:01 | C*06:02:01 | 62 |
| LSPRWYFYY,SPRWYFYYL | A*02:01 | B*07:02 | C*07:02 | 578 |
|  | A*03:01:01 | B*07:02:01 | C*07:02:01 | 401 |
|  | A*03:01 | B*07:02 | C*05:01 | 377 |
|  | A*01:01:01 | B*07:02:01 | C*07:01:01 | 334 |
|  | A*02:01:01 | B*07:02:01 | C*04:01:01 | 210 |

| **Supplementary Table 5: Variance estimate of the performance metrics for Pep-TCR Net on OO+J8D Prediction Dataset** | | | | | | |
| --- | --- | --- | --- | --- | --- | --- |
|  | Weighted OvR AUC | Macro OvR AUC | Test Accuracy | Weighted F1 Score | Weighted Precision | Weighted Recall |
| PE | 2.57E-08 | 5.43E-08 | 3.16E-07 | 3.49E-07 | 4.23E-07 | 3.16E-07 |
| ED | 9.55E-08 | 1.99E-07 | 8.29E-07 | 7.83E-07 | 1.14E-06 | 8.29E-07 |
| Tessa | 1.63E-08 | 3.94E-08 | 6.16E-07 | 7.37E-07 | 1.01E-06 | 6.16E-07 |
| PE + NE | 9.52E-08 | 1.24E-07 | 9.33E-07 | 7.97E-07 | 8.47E-07 | 9.33E-07 |
| ED + NE | 3.45E-07 | 2.56E-07 | 4.47E-07 | 5.27E-07 | 6.22E-07 | 4.47E-07 |
| Tessa + NE | 6.25E-08 | 1.24E-07 | 3.51E-07 | 3.79E-07 | 4.23E-07 | 3.51E-07 |
| PE + HLA | 1.11E-07 | 2.33E-07 | 1.04E-07 | 1.07E-07 | 1.07E-07 | 1.04E-07 |
| ED + HLA | 2.45E-07 | 2.89E-07 | 3.50E-07 | 3.91E-07 | 4.28E-07 | 3.50E-07 |
| Tessa + HLA | 4.69E-08 | 1.92E-07 | 8.91E-08 | 8.91E-08 | 1.09E-07 | 8.91E-08 |
| PE + VJ | 6.72E-08 | 8.90E-08 | 5.42E-07 | 5.78E-07 | 6.39E-07 | 5.42E-07 |
| ED + VJ | 1.36E-07 | 3.12E-07 | 4.47E-07 | 4.87E-07 | 5.91E-07 | 4.47E-07 |
| Tessa + VJ | 2.35E-08 | 2.32E-08 | 3.34E-07 | 3.14E-07 | 3.37E-07 | 3.34E-07 |
| PE + HLA + NE + VJ | 2.14E-07 | 1.84E-07 | 1.33E-07 | 1.49E-07 | 1.53E-07 | 1.33E-07 |
| ED + HLA + NE + VJ | 3.84E-07 | 4.34E-07 | 3.63E-07 | 4.09E-07 | 4.10E-07 | 3.63E-07 |
| Tessa + HLA + NE + VJ | 1.13E-07 | 1.31E-07 | 1.93E-07 | 1.96E-07 | 2.17E-07 | 1.93E-07 |
